# High-throughput single-cell transcriptomics reveals the female germline differentiation trajectory in *Arabidopsis thaliana*

**DOI:** 10.1038/s42003-021-02676-z

**Published:** 2021-10-01

**Authors:** Zhimin Hou, Yanhui Liu, Man Zhang, Lihua Zhao, Xingyue Jin, Liping Liu, Zhenxia Su, Hanyang Cai, Yuan Qin

**Affiliations:** 1 College of Life Sciences, State Key Laboratory of Ecological Pest Control for Fujian and Taiwan Crops, Fujian Provincial Key Laboratory of Haixia Applied Plant Systems Biology, College of Plant Protection, Fujian Agriculture and Forestry University, 350002 Fuzhou, China; 2 State Key Laboratory for Conservation and Utilization of Subtropical Agro-Bioresources, Guangxi Key Lab of Sugarcane Biology, College of Agriculture, Guangxi University, 530004 Nanning, China

**Keywords:** Cell fate, Oogenesis, Plant embryogenesis

## Abstract

Female germline cells in flowering plants differentiate from somatic cells to produce specialized reproductive organs, called ovules, embedded deep inside the flowers. We investigated the molecular basis of this distinctive developmental program by performing single-cell RNA sequencing (scRNA-seq) of 16,872 single cells of *Arabidopsis thaliana* ovule primordia at three developmental time points during female germline differentiation. This allowed us to identify the characteristic expression patterns of the main cell types, including the female germline and its surrounding nucellus. We then reconstructed the continuous trajectory of female germline differentiation and observed dynamic waves of gene expression along the developmental trajectory. A focused analysis revealed transcriptional cascades and identified key transcriptional factors that showed distinct expression patterns along the germline differentiation trajectory. Our study provides a valuable reference dataset of the transcriptional process during female germline differentiation at single-cell resolution, shedding light on the mechanisms underlying germline cell fate determination.

## Abstract (web summary)

Zhimin Hou, Yanhui Liu et al. used single cell RNA-seq to analyze the model organism, *Arabidopsis thaliana*, at three stages during female germline differentiation. They reconstructed the continuous trajectory of female germline differentiation, providing a valuable reference for future investigation of germline cell fate determination in *A. thaliana*.

## Introduction

Germline specification and differentiation are critical processes in sexual organisms. In flowering plants, germline cells are specified from somatic cells in the reproductive organs, the ovules, and anthers. In *Arabidopsis thaliana*, the female germline cell is initiated from a single sub-epidermal cell in the distal nucellus to form the archesporial cell (AC), which is morphologically distinguishable from the neighboring somatic cells by its larger size and conspicuous nucleus. The AC further elongates longitudinally and differentiates into the megaspore mother cell (MMC). The MMC then undergoes meiosis to generate megaspores and ultimately gives rise to a female gametophyte after degeneration of three megaspores, free nuclear mitotic divisions, and polarized cellularization^1^.

The molecular basis of female germline development in flowering plants is less well-understood than male germline development. Although there has been some recent progress^2^, the low abundance and general inaccessibility of female germline cells still greatly hamper the elucidation of the molecular mechanisms controlling female germline development. Previous gene profiling studies using bulk RNA sequencing (RNA-seq) analysis of ovule tissue or single-cell-type RNA-seq in combination with laser-assisted microdissection or fluorescence-assisted cell-sorting techniques to isolate MMC have helped reveal the transcriptional network underlying MMC specification and differentiation^3–5^. Although informative, these studies could not provide a comprehensive characterization of the continuous process of female germline cell differentiation.

The emerging high-throughput technology of single-cell RNA sequencing (scRNA-seq) enables broad gene profiling of thousands of single cells in a population, reflecting the heterogeneous nature of different cell types and the biological complexity of individual tissues. In animal systems, scRNA-seq has been successfully used to identify the entire cellular and molecular differentiation trajectory of certain cells and to characterize the transcriptomes of cell lineages during tissue development^6^. Recently, single-cell transcriptome profiling has also been established in the field of plants^7,8^. This technology has been applied in roots cells^9–14^, sperm cells^15^, mature female gametophytes^16^, vegetative shoot apex^17^, stomatal cell lineage^18^, isolated maize (*Zea mays*) male germline cells^19^, maize shoot stem cell^20^, rice root’s cells^21,22^, and captured moss (*Physcomitrella patens*) leaf cells^23^.

In this study, we analyzed the transcriptomes of 16,872 single cells from *Arabidopsis* ovule primordia across three developmental time points to investigate the transcriptional composition of different cell types present during early female germline development. Our study provides an unsupervised classification of cell populations and describes the extent of heterogeneity at the molecular level among populations of different cell types in ovule primordia undergoing germline differentiation, revealing continuous, dynamic patterns of change in gene expression during female germline initiation and specification.

## Results

### scRNA reveals ovule-primordium-specific expression patterns

**Fig. 1:**
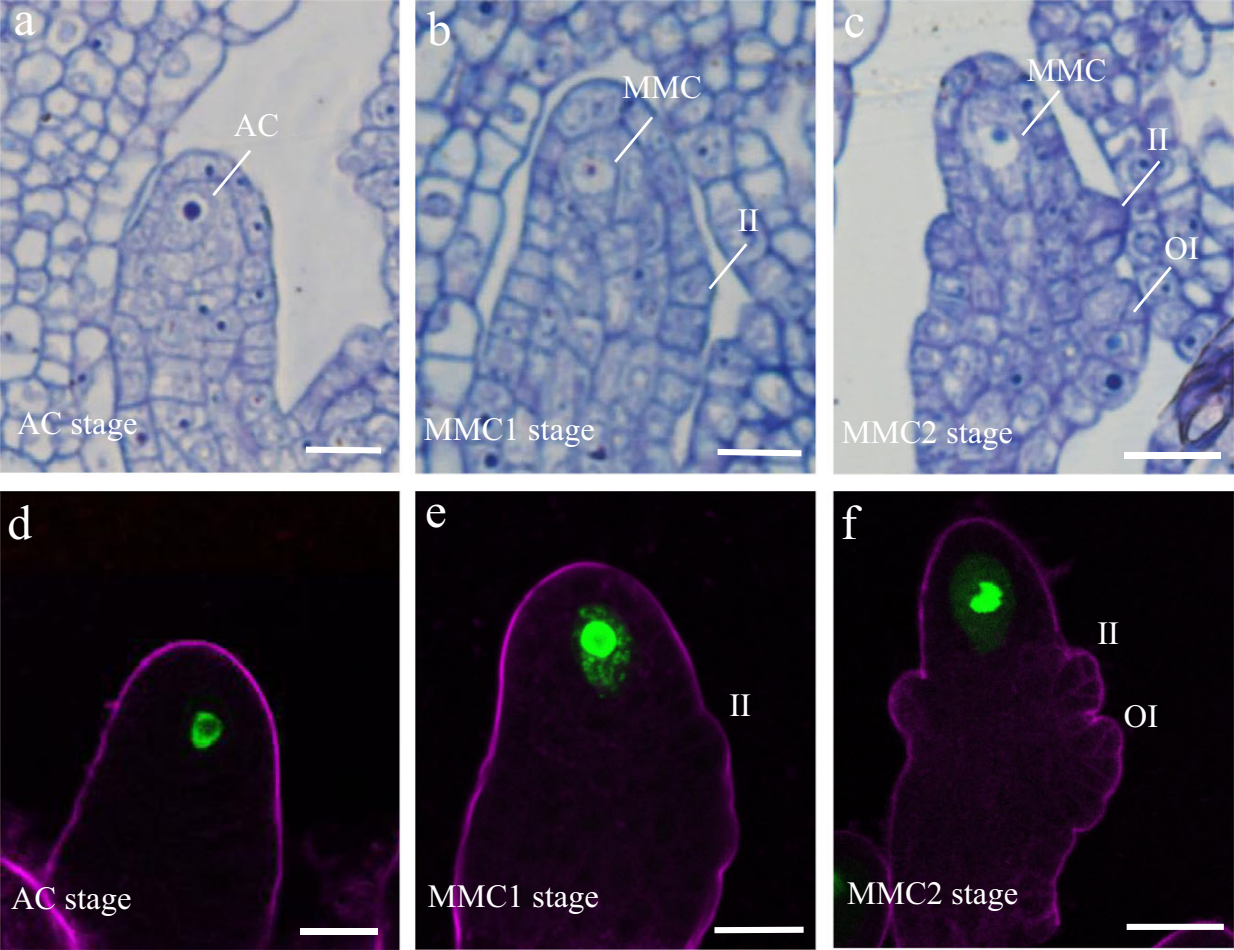
Ovule primordia structure at the AC, MMC1, and MMC2 stages. **a**–**c** Resin semi-thin sectioning of ovule primordia at the archesporial cell (AC; **a**) and megaspore mother cell stage 1 (MMC1; **b**) and MMC2 (**c**) stage. **d**–**f** *pKNU:KNU-Venus* expression patterns in ovule primordia at the AC (**d**), MMC1 (**e**), and MMC2 (**f**) stages. The magenta signal corresponds to FM4-64 dye outlining the ovule. II inner integument, OI outer integument. Scale bars, 10 μm.

To obtain single-cell expression profiles of *Arabidopsis* ovule primordia undergoing germline specification, we collected ovule primordia with placentas at developmental stages 2-I, 2-II, and 2-III from stage 9–10 flower buds of the MMC marker line *pKNU:KNU-Venus*^24^ and used these to generate protoplasts for 10Χ Genomics analysis (Supplementary Fig. 1). We performed resin semi-thin sectioning and examined the initiated AC and developing MMC in the ovule primordia by light microscopy (Fig. 1a–c). We also examined the expression of *pKNU:KNU-Venus* in the ovule primordia by confocal laser-scanning microscopy (CLSM) (Fig. 1d–f). We defined the stage 2-I sample, corresponding to ovule primordia at the AC stage, based on the presence of an enlarged AC and the absence of integument initiation (Fig. 1a). CLSM observation showed that at this stage 20.5% (*n* = 361) of ovules had started to express *KNU* (Fig. 1d), indicating that germline cell fate initiates at this stage. Stage 2-II samples comprised ovules that have just initiated inner integument development (Fig. 1b), of which 85.3% (*n* = 380) expressed *pKNU:KNU-Venus* (Fig. 1e). In stage 2-III ovules, outer integument development is initiated and the inner integument is composed of two cell layers (Fig. 1c), and 93.06% (*n* = 354) of these ovules expressed *pKNU:KNU-Venus* (Fig. 1f). We, therefore, defined these two sample types as containing ovule primordia at the MMC1 and MMC2 stages, respectively.

We captured 4577, 5054, and 7241 cells in the AC, MMC1, and MMC2-stage ovule-primordium samples, respectively, for single-cell RNA-seq library construction and sequencing and obtained 192,137, 160,835, and 135,514 mean reads per cell in the filtered dataset (Supplementary Data 1). The median unique molecular identifier (UMI) per cell in AC, MMC1, and MMC2-stage ovules were 4352, 2410, and 4096, respectively, which correspond to the expression of a median of 2322, 1526, and 2200 genes per cell, and a total of 24,165, 24,225, and 24,959 genes detected in the three samples, respectively (Supplementary Data 1 and Supplementary Fig. 2a–i). These values are comparable to that of reported scRNA-seq analysis of other tissues in *Arabidopsis*.

### MMC marker expression defines germline-associated gene clusters

**Fig. 2:**
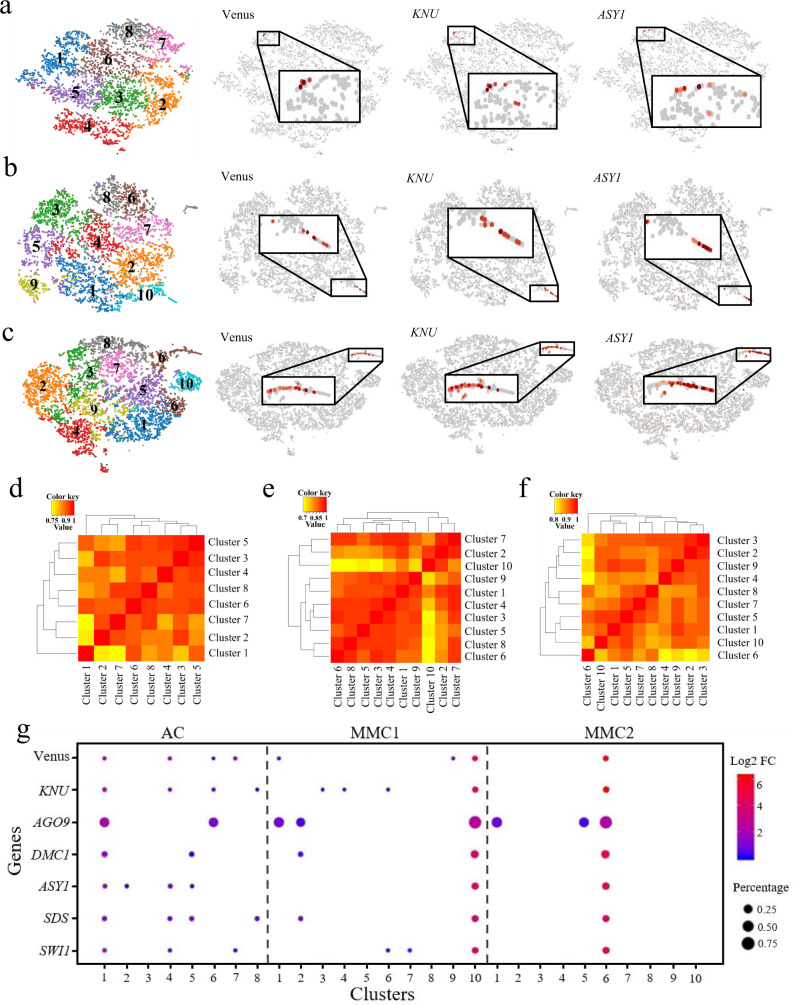
scRNA-seq data from ovule primordia at the AC, MMC1, and MMC2 stages. **a**–**c** t-SNE visualization of cells from ovule primordia at the AC (**a**), MMC1 (**b**), and MMC2 (**c**) stages. Left, eight, ten, and ten cell clusters (denoted by colors and numbers) were identified from the AC, MMC1, and MMC2-stage samples, respectively. Right, expression profiles of three selected MMC marker genes in each sample. The enriched expression in the subpopulation is magnified in each case. **d**–**f** Pearson correlation coefficient analyses of the cell clusters in the AC (**d**), MMC1 (**e**), and MMC2 (**f**) ovule samples. The value is Pearson correlation coefficient. The yellow-to-red color bar represents the Pearson correlation coefficient from low to high. **g** Expression of known marker genes in the cell clusters in the three samples. Dot size represents the proportion of cells in each cluster expressing a given gene. Color represents the relative expression level (Log2 FC) of the gene.

To identify distinct cell populations based on the genome-wide transcriptome profiles, we performed graph-based clustering of each sample. The unsupervised clustering was then projected onto a t-distributed stochastic neighbor-embedding (t-SNE) analysis. The t-SNE plots revealed a heterogeneous distribution of eight, ten, and ten cell clusters in AC, MMC1, and MMC2-stage ovule samples, respectively (Fig. 2a–c and Supplementary Data 2). The cell numbers of each cell cluster in the three samples ranged from 380–727, 235–842, and 357–1061, respectively (Supplementary Data 2), indicating there were differences in the size of each cell cluster within the sample. In order to verify whether different dimensional reduction techniques produce similar cell clusters, the uniform manifold approximation and projection (UMAP) algorithm was also employed to visualize the cell clusters in ovule samples at AC, MMC1, and MMC2 stages. Similarly, eight, ten, and ten cell clusters were identified with UMAP (Supplementary Fig. 2j–l and Supplementary Data 2). Since the three samples are from the same type of tissues at continuous developmental stages, we expected that there would be some similarity among the three samples. Considering that the clustering clouds of the three samples generated by t-SNE techniques are more similar to each other than that of UMAP, which indicates the local structure is better preserved by t-SNE, we used the t-SNE method for data visualization. We then performed Pearson correlation coefficient analysis for subpopulation correlation to determine the reliability of the cell clustering. The subpopulation correlation heatmap results indicated that cluster 1, cluster 10, and cluster 6 in the AC, MMC1 and MMC2-stage ovule samples, respectively, had the least correlation with other clusters among the samples (Fig. 2d–f), and were thus the most distinct clusters in each sample.

To identify the clusters most closely associated with germline cells in the three samples, we examined the expression of the Venus signal from the *pKNU:KNU-Venus* marker line. Expression was enriched mainly in cluster 1 of AC-stage ovule, cluster 10 of MMC1-stage ovule, and cluster 6 of MMC2-stage ovule samples (Fig. 2a–c). Moreover, *KNU* and *ASY1*, which are specifically expressed in the MMC in ovules^25^, were also mainly enriched in cluster 1 of AC-stage ovule, cluster 10 of MMC1-stage ovule, and cluster 6 of MMC2-stage ovule samples (Fig. 2a–c). We, therefore, defined these three clusters, designated as the AC.1, MMC1.10, and MMC2.6 cell clusters, respectively, as female germline-related cell clusters. There were 727 cells, 235 cells, and 659 cells in these three cell populations, accounting for 16%, 5%, and 9% of total cells in the AC, MMC1, and MMC2 stage of ovule samples, respectively (Supplementary Data 2). To further confirm the identity of these cell clusters, we examined the expression of known MMC marker genes, such as *AGO9*, *DMC1*, *SDS,* and *SWI1/DYAD*^26–30^, in the different clusters of the three samples. We observed that these were mostly enriched in the AC.1, MMC1.10, and MMC2.6 subclusters (Fig. 2g and Supplementary Data 3), further confirming the association of these clusters with female germline development.

### Identification of feature genes with cluster-specific expression patterns

We then determined the most uniquely expressed genes in each cell cluster in the three samples, by comparing each cluster with all other clusters within each sample to identify the genes that were most highly expressed in each cluster. The top 20 most discriminatory genes for each cluster were defined as the top 20 feature genes in each cluster for the three samples (Supplementary Data 4). To further confirm the clustering results and investigate the expression pattern of the feature genes of each cluster, we performed clustering analysis and generated a heatmap for the top 20 feature genes of each cluster (Supplementary Figs. 3–5). Most of the feature genes of each cluster were grouped together, confirming the accuracy of the clustering results.

The feature genes of the early germline AC.1 cluster include *WOX9*, *CEL2*, *TUBA4,* and *RPL11C* (Supplementary Data 4 and Supplementary Fig. 3); those of the MMC1.10 cluster include *AT4G29030*, *ERL1*, *RBG4*, and *AGO9* (Supplementary Data 4 and Supplementary Fig. 4); and those of the MMC2.6 cluster include *TUBA4*, *RBG7*, *HMGB6*, and *AT4G29030* (Supplementary Data 4 and Supplementary Fig. 5). Common enriched Gene Ontology (GO) terms among the feature genes of these germline-associated clusters (*P* < 0.05) included “protein metabolic process”, “peptide biosynthetic process”, “peptide metabolic process”, and “microtubule-based process” (Supplementary Data 5). These results suggest that genes involved in biogenesis and metabolism are highly active in germline-associated cell clusters.

### Subclusters associated with the female germline and surrounding soma

**Fig. 3:**
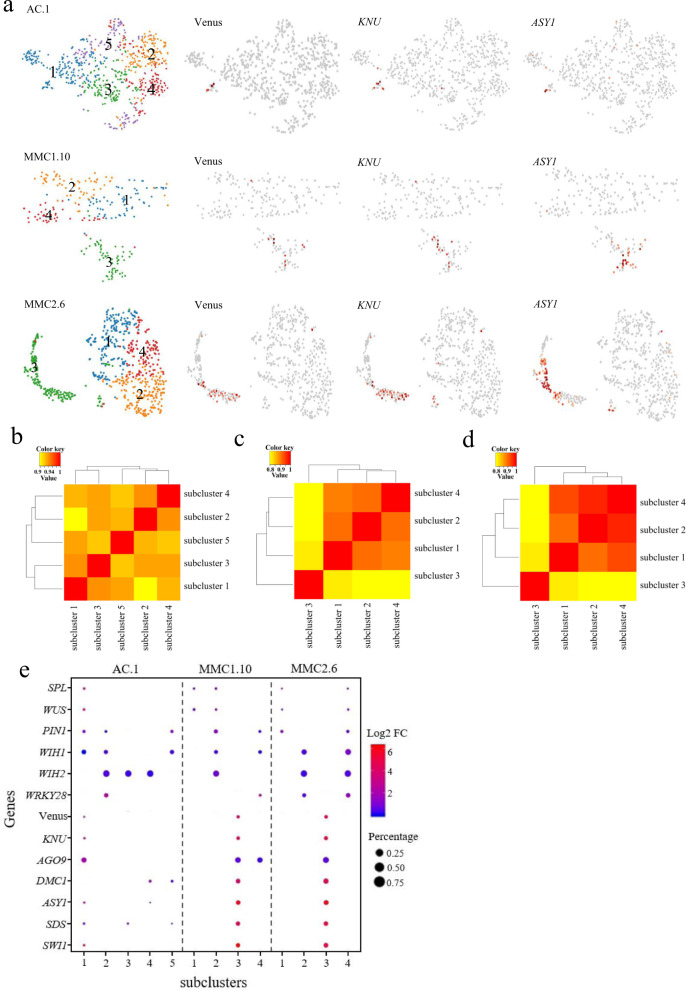
Identification of subclustering of the female germline-associated cell clusters. **a** t-SNE visualizations of cells from the AC.1, MMC1.10, and MMC2.6 clusters. Left, five, four, and four subclusters (denoted by colors and numbers) were identified from the three clusters, respectively. Right, expression profiles of selected MMC marker genes in each sample. **b**–**d** Pearson correlation coefficient analyses of the subcluster correlation in the AC.1 (**b**), MMC1.10 (**c**), and MMC2.6 (**d**) cell clusters. The value is Pearson correlation coefficient. The yellow-to-red color bar represents the Pearson correlation coefficient from low to high. **e** Expression of marker genes in the subclusters of the AC.1, MMC1.10, and MMC2.6 cell clusters. Dot size represents the proportion of cells in each cluster expressing a given gene. Color represents the relative expression level (Log2 FC) of the given gene.

Because the placenta tissue was included in the ovule samples, the female germline-associated cell clusters accounting for 5–16% of total cells in the three ovule samples, which is higher than the percentage of reproductive cells in the ovule sample, must contain other cell types in addition to the germline cells. To separate cell types within the female germline-associated cell clusters, we conducted a subclustering analysis at the three developmental time points (AC.1, MMC1.10, and MMC2.6). This identified five, four, and four subclusters in the AC.1, MMC1.10, and MMC2.6 clusters, respectively (Fig. 3a, Supplementary Data 2, and Supplementary Fig. 6). The subpopulation correlation heatmap showed that subcluster 1, subcluster 3, and subcluster 3 in the AC.1, MMC1.10, and MMC2.6 clusters, respectively, had the least correlation with other subclusters (Fig. 3b–d). *Venus* and *KNU* were predominantly expressed in these subclusters (Fig. 3a, e and Supplementary Data 3), confirming that these subclusters were associated with female germline cells. Feature genes of the female germline-associated subcluster at the AC stage included the known gene *AGO9* and unknown genes such as *AT1G05550*, *LTP6*, *PDF1*, and *AT4G29030* and were enriched for biological processes such as “translation”, “gene expression”, and “peptide biosynthetic and metabolic processes” (Supplementary Figs. 7 and 8 and Supplementary Data 6–8). Feature genes of the female germline-associated subcluster at the MMC1 stage included the known gene *DMC1* and unknown genes such as *AT5G43830, ACA10, PGDH1*, *AT1G70185,* and *ACT8*, and they were enriched for “ATP metabolic process” and “ion transport” (Supplementary Figs. 9 and 10 and Supplementary Data 6–8). Feature genes of the female germline-associated subcluster at the MMC2 stage included the known genes *DMC1*, *ASY1*, *ASY3,* and *MND1* and unknown genes such as *HIPP01*, *PAB7*, *AT1G68200*, *AT4G13710,* and *CALS5* and were enriched for “peptide transport”, “protein transport”, “chromosome organization” and “microtubule-based process” (Supplementary Figs. 11 and 12 and Supplementary Data 6–8). These results indicated that the germline characteristics are associated with active peptides biosynthesis and metabolism, ion transport, and chromosome organization.

Next, we used additional known markers to annotate other subclusters. Previous studies indicated that the positive germline regulator *SPOROCYTLESS/NOZZLE* (*SPL/NZZ*) and its downstream target gene *WUSCHEL* (*WUS*) are expressed in the epidermal cell layer of the nucellus cells^31,32^. *SPL* and *WUS* expression were limited to the cells in subcluster 1 of AC.1, subclusters 1 and 2 of MMC1.10, and subcluster 1 and 4 of MMC2.6 (Fig. 3e and Supplementary Fig. 13), suggesting that subcluster 1 of AC.1 may represent the origin of the germline and epidermal cell lineage, and subclusters 1 and 2 of MMC1.10 and 1 and 4 of MMC2.6 may contain the epidermal nucellus cells surrounding the MMC. In agreement with this view, another gene expressed in epidermal nucellus cells, *PIN1*^33^, was also detected mainly in subcluster 1 of AC.1, subcluster 2 of MMC1.10, and subclusters 1 and 4 of MMC2.6 (Fig. 3e and Supplementary Fig. 13).

Feature genes of the nucellar epidermis cell subclusters included *ENODL14*, *AT2G27385*, *KN, AT5G16250*, and *WOX9* and were enriched for the GO terms “photosynthesis”, “RNA biosynthetic process”, and “regulation of transcription” (Supplementary Figs. 7, 9, 11, and 13 and Supplementary Data 9). We detected two downstream target genes of *WUS*, *WIH1*, and *WIH2*, which are reported to be specifically expressed in the distal nucellus and to act to promote germline cell-fate transition from somatic precursor cells^34^, in most subclusters of AC.1, but their expression was concentrated in subclusters 2 of MMC1.10 and 4 of MMC2.6, respectively (Fig. 3e). In addition, *WRKY28*, which was specifically expressed in the cells in the sub-epidermal cell layers surrounding the MMC^35^, was mainly detected in subcluster 2 of AC.1, subcluster 4 of MMC1.10, and subclusters 2 and 4 of MMC2.6 (Fig. 3e). These results suggested that in comparison with the subclusters of MMC1.10 and MMC2.6, the cells in the different subclusters of AC.1 may be more transcriptionally similar to each other; moreover, subcluster 4 of MMC1.10 and subclusters 2 and 4 of MMC2.6 may comprise the sub-epidermal nucellus cells surrounding the MMC.

Feature genes of the subclusters of sub-epidermal nucellus cells surrounding the MMC included *DOF5.3*, *ATPAP1*, *ROPGEF1*, *ML3*, *AT3G24420*, and *AT3G15630* and were enriched for the GO terms “lipid biosynthetic process”, “lipid metabolic process” and “response to stress” (Supplementary Figs. 7, 9, 11, and 14 and Supplementary Data 9). Notably, the epidermal nucellus markers (*SPL*, *WUS*) and sub-epidermal nucellus marker (*WRKY28*) were coexpressed in subcluster 4 of MMC2.6, suggesting a degree of commonality between this subset of nucellus epidermis and the sub-epidermis. Together, these findings suggested the successful capture of the main cell types in the epidermal and sub-epidermal cell layers, including the female germline and its surrounding soma, and revealed the molecular characteristics of these cell types, illustrating the efficacy of scRNA-seq for studying rare cell types within a tissue.

### Reconstruction of the MMC differentiation trajectory in pseudotime

**Fig. 4:**
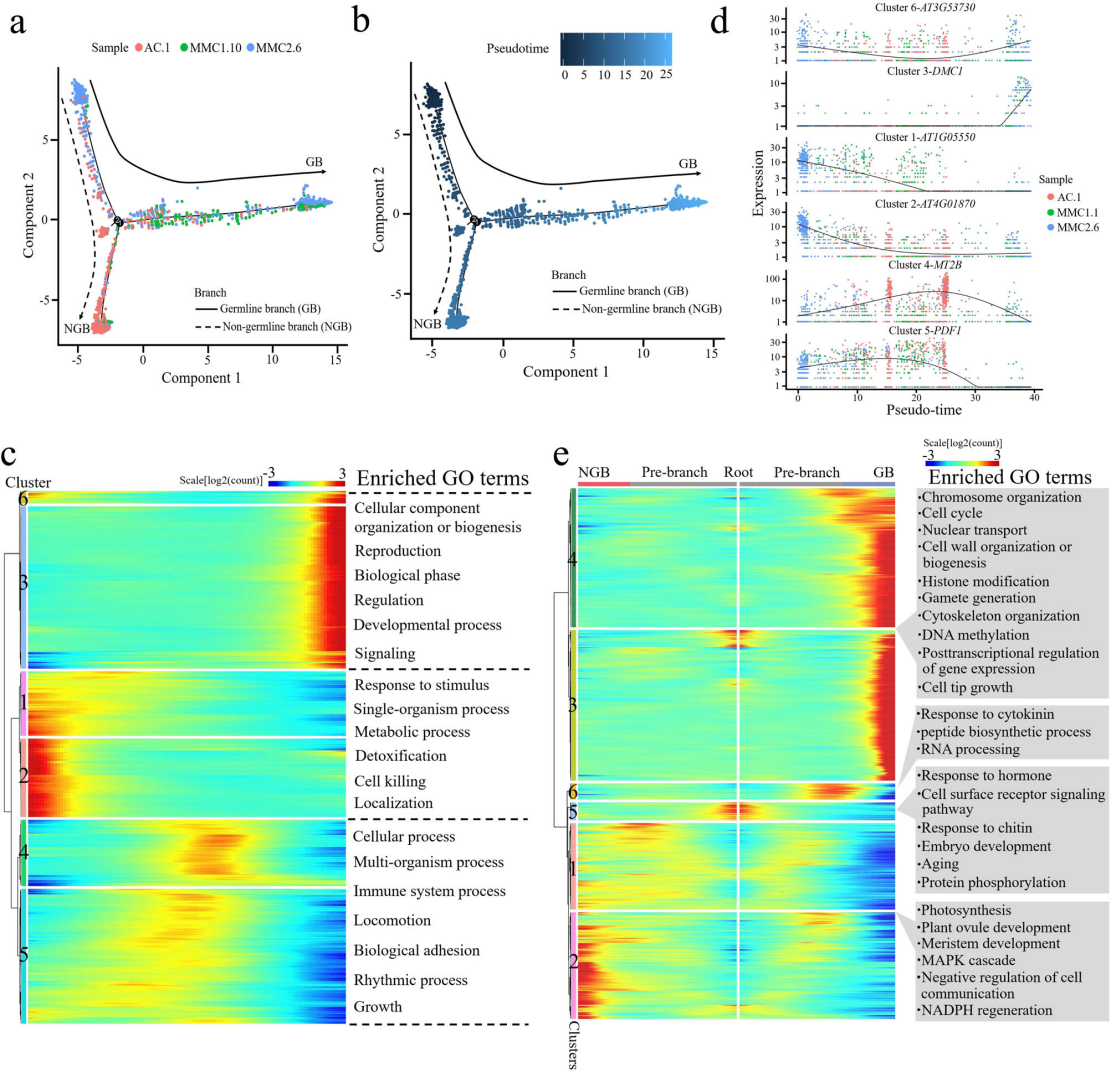
Reconstruction of MMC differentiation trajectory in a pseudotime manner. **a**, **b** Pseudotime trajectory of MMC differentiation. The horizontal and vertical coordinates are two principal components, and the dots represent different cells, with different colors representing the sample identity (**a**) or the pseudotime (**b**). Color from darkest to lightest blue in (**b**) represents pseudotime from beginning to end. The black circles represent the different branch points, and the solid and dashed lines represent the trajectories of the germline branch (GB) and non-germline branch (NGB), respectively. **c** Expression heatmap of 1738 DEGs across pseudotime. The horizontal coordinate represents quasi-chronological order, and the vertical coordinate represents one gene per row. Rows were grouped based on similarity of gene expression, resulting in the six clusters indicated at the left. The color bar indicates the relative expression level. Representative GO terms are listed at the right. **d** Expression values of a representative gene in each cluster along the pseudotime axis. The abscissa represents the quasi-chronological order, and the ordinate represents the relative expression value of genes. The black line denotes the smoothed average expression. **e** Branched heatmap showing 4020 DEGs with branch-specific expression patterns in pseudotime. The root of the tree is in the middle of the plot, and expression from the earliest cells to the non-germline cells of the NGB is shown progressing to the left, whereas the germline cell progression in the GB is shown progressing to the right of the root. The color bar indicates the relative expression level. Representative GO terms are listed at the right.

To infer the developmental trajectory of female germline differentiation, we conducted a pseudotime analysis of the AC.1, MMC1.10, and MMC2.6 cell clusters. The analysis showed that a number of cells from the AC.1 and MMC2.6 clusters were assembled at the beginning of pseudotime (Fig. 4a, b). The majority of cells from AC.1 and MMC1.10 were concentrated in the center of the pseudotime trajectory, and the end of the trajectory included most of the MMC2.6 cells and the smallest proportion of AC.1 cells (Fig. 4a, b). The reconstructed pseudotime trajectory revealed a linear ordering of cells reflecting the arrangement of clusters and states during MMC differentiation. Every branch in the inferred trajectory comprised cells from all three clusters (Fig. 4a, b), suggesting that the clusters include some intermediate cells.

To explore the gene expression dynamics along the MMC differentiation trajectory, we performed a hierarchical clustering analysis of the identified 1738 differentially expressed genes (DEGs) across pseudotime. We identified six major categories of gene clusters in the characterized patterns (Fig. 4c and Supplementary Data 10). Genes in clusters 6 and 3 exhibited similar expression patterns, with gene expression gradually increasing over pseudotime and reaching a maximum at the final stage of the developmental program. These genes were largely involved in the biological processes “cellular component organization or biogenesis”, “reproduction”, “biological phase”, “regulation”, “developmental process”, and “signaling” (Fig. 4c and Supplementary Data 10). The expression levels of cluster 1 and 2 genes decreased continuously over pseudotime. These genes predominantly participated in “response to stimulus”, “single-organism process”, “metabolic process”, “detoxification”, “cell killing”, and “localization” (Fig. 4c and Supplementary Data 10). Expression levels of cluster 4 and 5 genes transiently increased and then finally decreased, implying that there were two temporary waves of transcription along the differentiation trajectory. These genes were enriched in “cellular process”, “multi-organism process”, “immune system process”, “locomotion”, “biological adhesion”, “rhythmic process”, and “growth” (Fig. 4c and Supplementary Data 10). The expression waves of the representative DEGs of the six gene clusters in single cells from the three samples across pseudotime are shown in Fig. 4d.

Next, we analyzed the expression changes of the top 100 DEGs and established that they were grouped into six major categories (Supplementary Fig. 15a). The expression waves of the top five DEGs across pseudotime are also indicated (Supplementary Fig. 15b). These results revealed the precise regulation of a vast number of individual gene expression changes during MMC differentiation.

### Identifying genes potentially involved in MMC differentiation

Over pseudotime, the trajectory of the ovule cells bifurcated (at what we defined as branchpoint 1) into two main branches: the germline branch (GB) and the non-germline branch (NGB) (Fig. 4a, b). We mapped the known germline markers *DMC1*, *ASY1*, *SDS*, and *AGO9* onto the pseudotime trajectory and confirmed that all four were expressed only in the GB (Supplementary Fig. 16). The occurrence of this branch node represented the somatic-to-germline cell-fate changes in the cells.

To elucidate the molecular dynamics that distinguished the two branches, we analyzed the expression data of the cells at the branch node in the pseudotime trajectory and identified the DEGs associated with the branching, using the Monocle 2 tool BEAM (branched expression analysis modeling)^36^. This analysis identified 4020 DEGs associated with branchpoint 1, which were classified into six groups (Fig. 4e and Supplementary Data 11). The majority of cluster 4 and 3 genes displayed increased expression along the GB trajectory and low expression in the NGB trajectory; these included the MMC marker genes *KNU*, *AGO9*, *DMC1*, *ASY1*, and *SDS*. These genes were enriched for the GO terms “chromosome organization”, “cell cycle”, “nuclear transport”, “cell wall organization or biogenesis”, “histone modification”, “gamete generation”, “cytoskeleton organization”, “DNA methylation”, “posttranscriptional regulation of gene expression”, and “cell tip growth” (Fig. 4e). Cluster 6 genes showed low expression in the pre-branch and NGB trajectories, but a sharp increase near the branchpoint followed by a decrease in the GB trajectory; they were predominantly involved in “response to cytokinin”, “peptide biosynthetic process”, and “RNA processing” (Fig. 4e). Cluster 5 genes were highly expressed at the root and underwent loss of expression as cells processed into either BG or NGB, and they were enriched for “response to hormone”, “cell surface receptor signaling pathway”, “response to chitin”, “embryo development”, “aging”, and “protein phosphorylation” (Fig. 4e). Cluster 1 and 2 genes displayed an increasing expression along the pre-branch and NGB trajectory but the reduced expression in the GB trajectory, with an overrepresentation of genes involved in “photosynthesis”, “plant ovule development”, “meristem development”, “MAPK cascade”, “negative regulation of cell communication”, and “NADPH regeneration” (Fig. 4e).

We also extracted the top 50 DEGs between the branches and grouped them into six clusters (Supplementary Fig. 17). The distinct branch-specific expression patterns of these DEGs may reflect their different roles or different times of action during the progression of germline cell-fate specification and differentiation.

### Identification of transcription factors coordinated with MMC differentiation

**Fig. 5:**
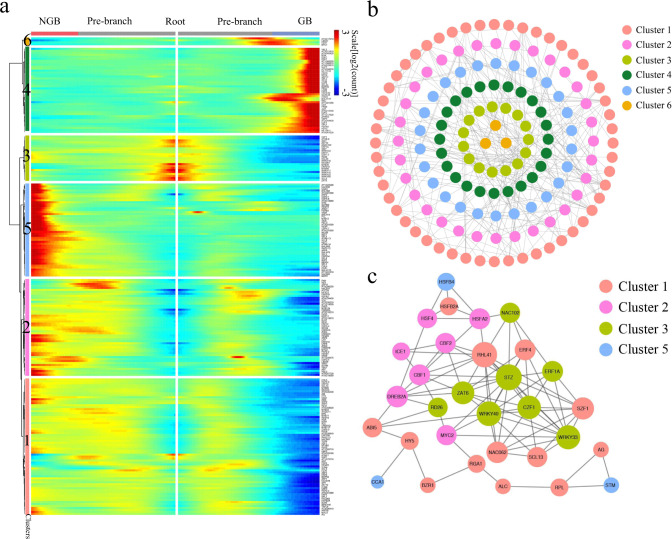
Transcription factor (TF) expression during MMC differentiation. **a** Branched heatmap showing 235 TFs with branch-specific expression patterns in pseudotime. The root of the tree is in the middle of the plot, and expression from the earliest cell to the non-germline cells of the NGB is shown progressing to the left, whereas the germline cell progression in the GB is shown progressing to the right of the root. **b** Correlation network of 153 TFs that are differentially expressed between branches with a degree cutoff of 1. TFs are colored according to their assignment in the gene clusters in (**a**). **c** The same correlation network with a degree cutoff of 6. Node size is equivalent to the number of predicted connections.

To elucidate further the regulatory control of the MMC differentiation program, we extracted transcription factor (TF) genes from the DEGs associated with the branching, resulting in 235 TFs. A heatmap analysis showed nonrandom expression patterns of these TF genes along the pseudotime trajectory starting from the root and continuing down the two branches (Fig. 5a and Supplementary Data 12). We identified four distinct patterns of TF gene expression along the trajectory: (1) low TF expression along the pre-branch and NGB trajectories but high expression along the GB trajectory (e.g., *KNU*, *E2FA*, *E2FB*, *E2FC*, and *CDC5*); (2) high expression at the root with loss of expression as cells progressed into either GB or NGB (e.g., *CZF1*, *ERF11*, *WRKY18*, *MYB96,* and *ZAT6*); (3) low TF expression along the pre-branch and GB trajectories with high expression along the NGB trajectory (e.g., *ARF2*, *STM*, *YAB1*, *WOX6*, and *HEC1*); and (4) increased expression in the pre-branch but reduced expression along the GB trajectory (e.g., *PAN*, *AG*, *STK*, *HY5,* and *BZR1*) (Fig. 5a). Expression profiles of the 235 TF genes showed expression waves along the developmental trajectory, suggesting an intricate regulation of germline cell-fate determination. The functions of some of the TFs in germline specification have been reported, including *KNU*, *AG*, *E2FA, E2FB*, and *E2FC*^24,37,38^. Most of the TFs we identified, however, have not previously been implicated in MMC differentiation. The distinct expression patterns of TFs upon cell-fate branching may reflect their potential roles in cell-fate progression, providing valuable information for the direction of future functional studies to identify the key components in female germline cell-fate determination.

To further elucidate the genetic coordination during female germline differentiation, we generated correlation networks of the differentially expressed TF genes associated with the branching. Figure 5b shows the correlations among TF genes. We then filtered the network down to its 31 core components and identified the highly connected central regulators in the MMC differentiation trajectory, the majority of which have not yet been assigned functions in MMC differentiation. Future research should investigate the functions of these core TF genes that showed distinct expression patterns during MMC development, which may imply that they have biological roles in this process. For example, the cluster 1 core TF genes (e.g., *RHL41*, *ERF4*, *ABI5*, and *NAC062*) displayed an expression peak as cells approached the branchpoint (Fig. 5a, c), indicating that, like the well-characterized *AG* (*AGAMOUS*)^39^, they could be the initiators of cell-fate specification, whereas the cluster 3 core TF genes (e.g., *STZ*, *WRKY33*, *CZF1*, *ERF1A*) showed a pattern of increased expression as the cells progressed into the GB (Fig. 5a, c), suggesting that they could be key players in germline cell development.

Together, these results depicted the sequential molecular dynamics associated with the somatic-to-germline cell-fate transition and revealed the key components of correlation networks, providing informative reference data for further study.

### Integrated analysis of the three single-cell transcriptomes by AGGR pipeline

**Fig. 6:**
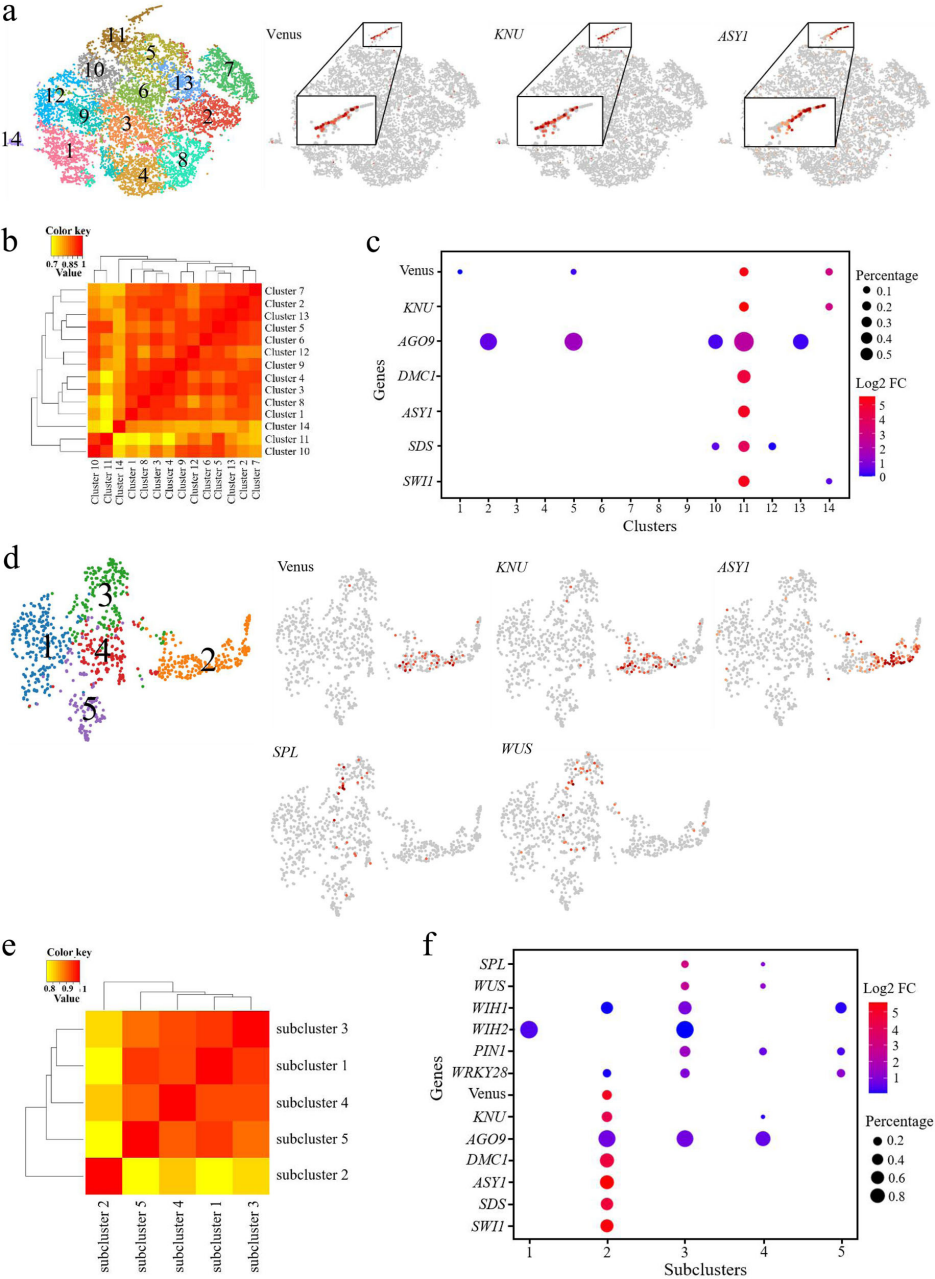
scRNA-seq Data of ovule primordia and Identification subclustering of the female germline containing cell clusters. **a** t-SNE visualization of cells from ovule primordia at AGGR. The left panel shows fourteen cell clusters were identified from the AC, MMC1, MMC2-stage samples. The right three panels indicate the expression profiles of selected MMC marker genes in each sample. The enriched expression in the subpopulation is magnified in each case. **b** Pearson correlation coefficient analysis for the cell clusters in AGGR. The value is the Pearson correlation coefficient. The yellow-to-red color bar represents the Pearson correlation coefficient from low to high. **c** Expression of known marker genes in the cell clusters. Dot size shows the proportion of cells in each cluster expressing a given gene. Color represents the relative expression level (Log2 FC) of the given gene. **d** t-SNE visualization of cells from the cluster 11. The left panel shows five subclusters that were identified from cluster 11. The right five panels indicate indicating the expression profiles of selected MMC marker genes and the integuments. **e** Pearson correlation coefficient analysis for the subcluster correlation in the cluster 11. The value is the Pearson correlation coefficient. The yellow-to-red color bar represents the Pearson correlation coefficient from low to high. **f** Expression of known marker genes in the cell clusters in the five subclusters. Dot size represents the proportion of cells in each cluster expressing a given gene. Color represents the relative expression level (Log2 FC) of the gene.

Finally, we integrated all the 16,872 cells from the three samples by Aggregating Multiple GEM Groups (AGGR) pipeline and normalized the data by equalizing the read depth among libraries. There were 134,240 mean reads per cell obtained in the post-normalized dataset (Supplementary Data 1 and Supplementary Fig. 18). The median number of UMI counts per cell was 3634, corresponding to a median of 2031 genes per cell. We then projected all single cells on a t-SNE plot and identified fourteen clusters with cell numbers ranged from 163 to 1849 in each cluster (Fig. 6a and Supplementary Data 2). The MMC marker genes *KNU*, *AGO9*, *DMC1*, *ASY1*, *SDS*, and *SWI1/DYAD* are all preferentially or specifically expressed in cluster 11, therefore cluster 11 was designated as the germline-associated cell cluster, which comprises 916 cells and accounts for 5% of the whole population (Fig. 6a, c and Supplementary Data 2 and 3). Subpopulation correlation analysis indicated that cluster 11 was the most distinct cluster among the sample (Fig. 6b).

Subclustering of the germline-associated cell cluster identified five subclusters (Fig. 6d and Supplementary Fig. 19). MMC marker gene expression and subpopulation correlation analysis revealed the specificity of subcluster 2 to female germline cells (Fig. 6e, f and Supplementary Data 3). Feature genes of the female germline subcluster 2 included the known genes *DMC1*, *ASY1, MND1*, and *TPD1*, and unknown genes such as *PAB7*, *AT1G68200*, *AT4G13710*, and *UGP* and were enriched for “chromosome organization”, “ribonucleotide metabolic process”, and “microtubule-based process” (Supplementary Figs. 20 and 21 and Supplementary Data 6–8). *WUS* and *SPL* are mainly detected in subcluster 3, which was assigned to the epidermal nucellus cells subcluster (Fig. 6d). Feature genes of the epidermal nucellus cells subcluster 3 include *CIPK14*, *ERL1*, and *ATL6*, and were enriched for the GO terms “cellular macromolecule metabolic process”, “translation” and “peptide metabolic process” (Supplementary Fig. 20 and Supplementary Data 6, 7, and 9). These results were largely consistent with the above findings from the analysis of the three individual samples.

**Fig. 7:**
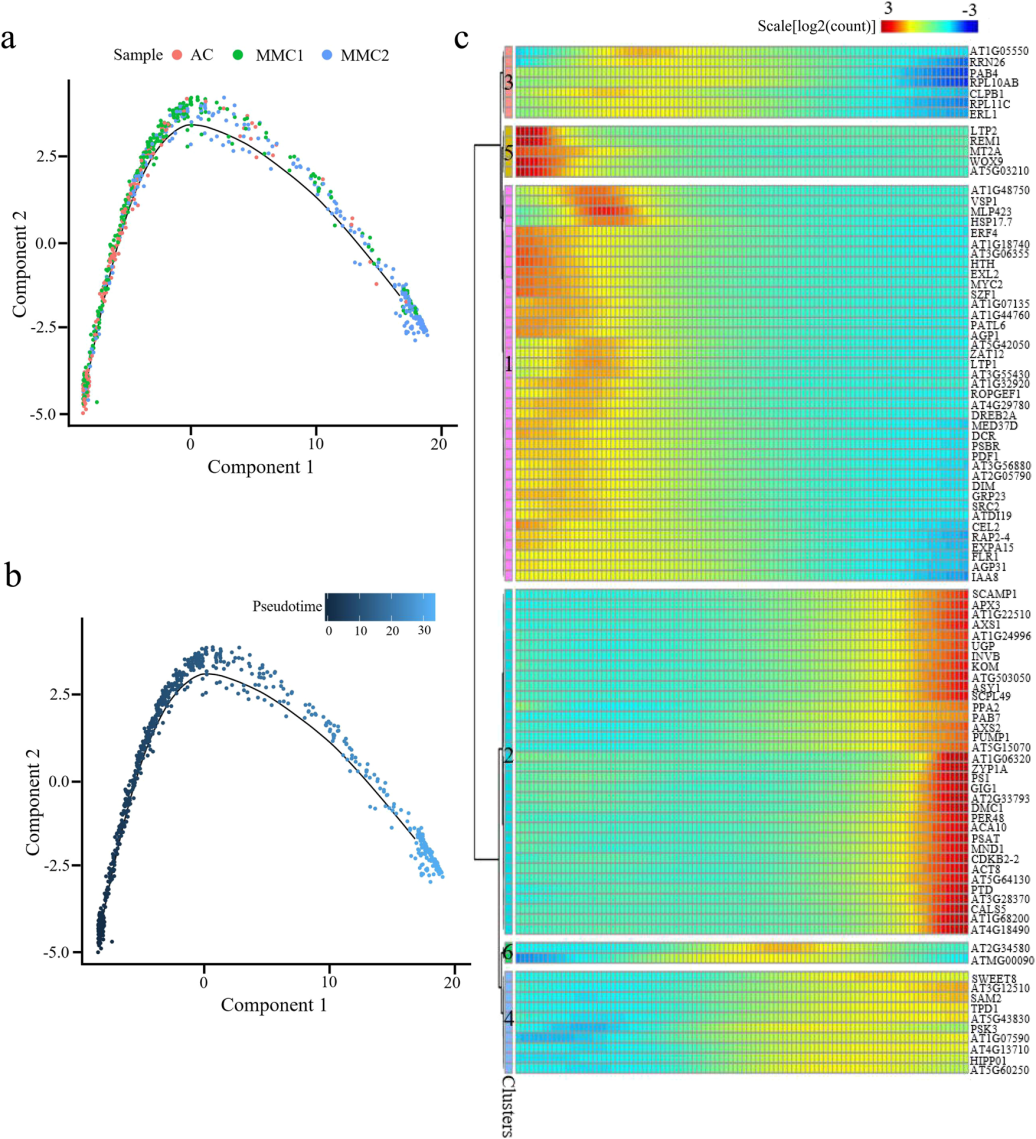
Reconstruction of the differentiation trajectory in a pseudotime manner. **a**, **b** Pseudotime trajectory of the cluster 11. The horizontal and vertical coordinates are two principal components, and the dots on the way represent different cells, in which different colors represent the sample identity (**a**) or the pseudotime (**b**). The color from darkest blue to lightest blue (**b**) represents pseudotime from beginning to end. **c** Expression heatmap of the top 100 DEGs across pseudotime. The abscissa is in quasi-chronological order, and the ordinate represents one gene per row. Each column represents the average expression value under the current cell state. Rows were grouped based on similarity in gene expression, resulting in six clusters indicated left. The color bar indicates the relative expression level.

A pseudotime analysis on cells in cluster 11 was conducted to order the developing germline cells in a putative developmental trajectory. The inferred trajectory showed that the majority of cells from the AC and MMC1 samples were concentrated at the beginning or the center of the trajectory (Fig. 7a, b). A small number of cells from the AC and MMC1 samples were assembled at the end of the reconstructed trajectory. In contrast, the cells from the MMC2 sample were mainly distributed at the center and the end of the trajectory (Fig. 7a, b). The inferred trajectory reflected the gradual differentiation of MMC from the early to the mature stage. To depict the gene expression dynamics across pseudotime, we generated an expression heatmap for the top 100 DEGs along the trajectory (Fig. 7c and Supplementary Data 13). Six distinct gene clusters were identified with cluster 1 and 5 genes showing reduced expression along the timeline and cluster 2 and 4 genes exhibiting increased expression along the timeline. Genes in clusters 3 and 6 were transiently upregulated and then downregulated along the timeline. These data demonstrated a continuous and dynamic differentiation process of MMC development and further reveal genes that may regulate or implicate in different developmental stages of MMC differentiation.

### Validation of marker gene expression pattern

**Fig. 8:**
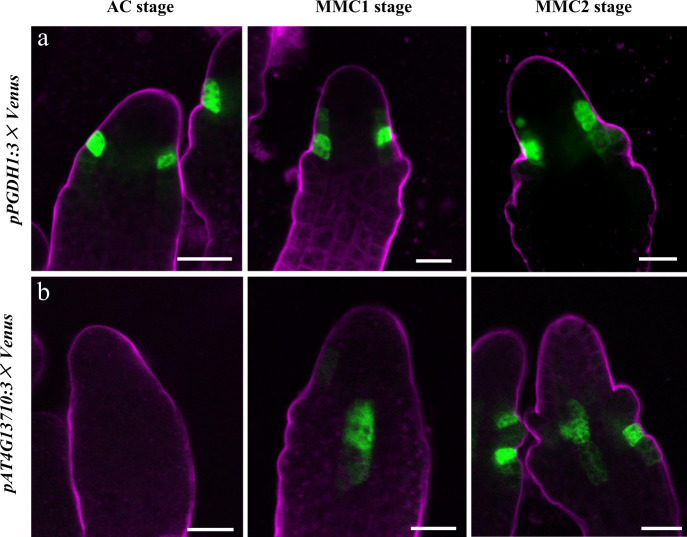
Expression patterns of *PGDH1* and *AT4G13710* in ovules during germline specification. **a**, **b***pPGDH1:3×Venus* signals (**a**) and *pAT4G13710:3× Venus* (**b**) examined in ovules from AC to MMC2 stages. The magenta signal corresponds to FM4-64 dye outlining the ovule. Scale bars, 10 μm.

We selected two genes, *PGDH1* and *AT4G13710*, identified as feature genes in female germline-associated subclusters at the MMC1 and MMC2 stages for validation analysis (Supplementary Figs. 9–12 and Supplementary Data 6–8). We generated the promoter–reporter lines *pPGDH1:3×Venus* and *pAT4G13710:3×Venus* to assess the spatiotemporal expression pattern of the feature genes. The results showed that *PGDH1* was specifically expressed in the epidermis of distal nucellus cells near the inner integument primordia from AC to MMC2 stages (Fig. 8a). In comparison, *AT4G13710* had different expression patterns during megasporogenesis. At the AC stage, the *pAT4G13710:3×Venus* signal was undetectable (Fig. 8b). At the MMC1 stage, *AT4G13710* was mainly expressed in the central region of the nucellus. The *AT4G13710* signal was also detected in epidermal cells in the integument region at the MMC2 stage (Fig. 8b). These two feature genes in female germline-associated subclusters express in specific cell types surrounding the female germline that have not been reported previously, indicating the power of scRNA-seq as a tool to uncover novel cell types. The expression patterns of *PGDH1* and *AT4G13710* suggest that the sporophytic cells surrounding the female germline may share close molecular features with the germline cells and frequent molecular exchange may occur between these cells.

**Fig. 9:**
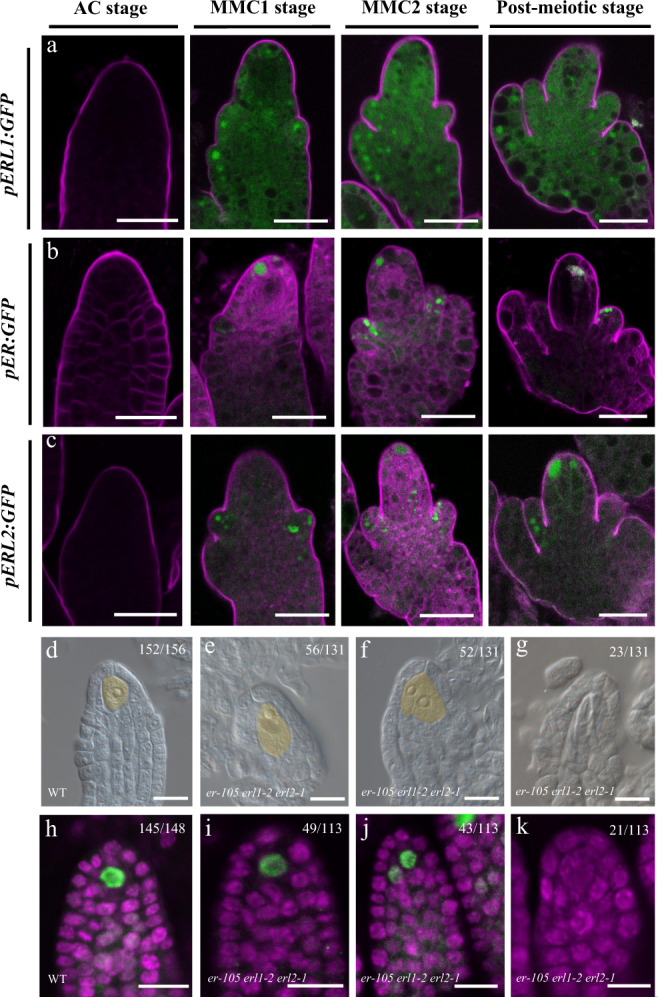
Expression patterns of *ERL1*, *ER,* and *ERL2* in ovules and MMC specification phenotype in *er-105 erl1-2 erl2-1* ovules. **a**, **b** GFP signals were detected in ovules at different stages from AC to the post-meiotic stages. Expression patterns of *pERL1*: *GFP* (**a**); expression patterns of *pER*: *GFP* (**b**); expression patterns of *pERL2*: *GFP* (**c**). The magenta signal corresponds to FM4-64 dye outlining the ovule. Scale bars,10 μm. **d**–**g** DIC images of wild-type (WT) (**d**) and *er-105 erl1-2 erl2-1* (**e**–**g**) ovules. Numbers denote the frequencies of the phenotypes shown. MMC is fake colored by yellow. Scale bar, 20 μm. **h–k** AGO9 immunolocalization of WT (**h**) and *er-105 erl1-2 erl2-1* (**i**–**k**) ovules. Green and magenta signals correspond to AGO9 localization and propidium iodide signal, respectively. Scale bars,10 μm.

We previously showed that *OsERECTA2* (*OsER2*) receptor-like kinase gene exhibits preferential expression patterns in AC and MMC stages of rice ovules and plays essential roles during female germline specification^40^. Here, *Arabidopsis ERECTA-LIKE 1* (*ERL1*), the homolog of *OsER2*, was identified as a feature gene of the MMC1.10 germline-associated gene cluster (Supplementary Fig. 4 and Supplementary Data 4) and a feature gene of the epidermal nucellus cell subcluster (Supplementary Fig. 20 and Supplementary Data 6, 7, and 9). To validate the marker gene expression pattern, we generated the promoter–reporter line *pERL1:GFP*. In AC-stage ovules, the *pERL1:GFP* signal was undetectable. From MMC1 to the post-meiotic stage, *pERL1:GFP* expression was preferentially detected in the epidermal nucellus cells near the integument region (Fig. 9a). In *Arabidopsis*, the *ERECTA* family consists of three genes: *ER*, *ERL1*, and *ERL2*, and they generally function redundantly^41,42^. Therefore, we generated *pER:GFP* and *pERL2:GFP* and found that *ER* and *ERL2* also display epidermal nucellus cells preferential expression patterns during megasporogenesis (Fig. 9b, c). *pER:GFP* and *pERL2:GFP* were not detectable in AC-stage ovule. In the MMC1 stage, *pER:GFP* was examined in the epidermis of the distal nucellus, while *pERL2:GFP* was located in the epidermis of the inner integument. In the MMC2 stage and post-meiotic stage of ovules, both *pER:GFP* and *pERL2:GFP* displayed preferentially expression in the epidermis of distal nucellus cells and the inner integument (Fig. 9c). The epidermal nucellus cell preferential expression pattern of the *ERECTA* gene family is consistent with the results of cell clustering analysis, implying that scRNA-seq is a powerful tool to identify potential important players in female germline differentiation.

### *ERECTA* gene family is involved in MMC specification

*ER* family genes play a redundant role in female gametophyte development and integument growth^43,44^. To determine whether *ER* family is involved in MMC differentiation, we generated *er-105 erl1-2 erl2-1* triple mutant. Differential interference contrast (DIC) microscopy analysis showed that 39.7% (*n* = 131) of *er-105 erl1-2 erl2-1* ovule displayed supernumerary enlarged MMC-like cells, which is significantly higher than the observed ~2.6% (*n* = 156) in wild-type ovules (*P* < 0.01 by *t* test, Fig. 9d–f). In addition, we also found that 17.6% (*n* = 131) of *er-105 erl1-2 erl2-1* ovule do not have MMC (*P* < 0.01 by *t* test, Fig. 9g), which is not observed in wild-type ovules (0%, *n* = 156). To determine whether the enlarged cells acquire MMC identity, we carried out whole-mount immunolocalization using anti-ARGONAUTE9 (AGO9) antibodies. AGO9 is only detected in the nucleus of MMCs in 98.0% (*n* = 148) of wild-type ovules (Fig. 9h) and serves as a marker for MMC^45^. We observed that in 38.1% (*n* = 113) of *er-105 erl1-2 erl2-1* ovule, AGO9 accumulated in the nuclei of more than one cell (Fig. 9j); 18.6% (*n* = 113) of *er-105 erl1-2 erl2-1* ovule, AGO9 signal was not detectable (Fig. 9k), consistent with DIC observation. These results suggest that *ER* family plays an important role in restricting MMC specification to a single cell and promoting MMC differentiation.

## Discussion

In plants, the female germline is buried in somatic cells within the ovule primordium, which in turn is located inside the gynoecium. The difficulty of isolating female germline cells, and the small number of ovules within an *Arabidopsis* flower bud, make it challenging to characterize female germline differentiation at the transcriptome level^46^. Here, we report the successful application of scRNA-seq to reveal the developmental dynamics of plant female germline cells and provide the high-resolution reference dataset of single-cell transcriptomes during female germline specification and differentiation in *Arabidopsis*.

In this study, we obtained a total of 16,872 single-cell transcriptomes from *Arabidopsis* ovule primordia at three developmental stages during MMC specification. Making use of MMC marker genes, we identified AC.1, MMC1.10, and MMC2.6 three clusters as germline-associated cell clusters. Subclustering analysis of these clusters further identified the germline subclusters and the neighbor soma cell subclusters. Because of the paucity of well-characterized cell markers in ovule primordia undergoing megasporogenesis, further work will be required to identify unknown cell types and reveal the cell identities in other cell populations identified here.

Our clustering and subclustering analyses revealed heterogeneity among the cell types in ovule primordia during germline specification. This heterogeneity allowed us to correlate distinct gene expression signatures with different subclusters. The feature genes of the female germline subclusters from the AC to MMC2 stages were enriched for “gene expression” and “macromolecule biosynthetic and metabolic processes”, suggesting that tremendous numbers of genes and proteins are activated during germline development. In line with the important roles of peptide-mediated cell communication in germline specification^34,47^, enriched GO terms related to peptide biosynthesis and transport were identified in germline subclusters. At the MMC2 stage, the germline cell subcluster showed enrichment for genes linked to “chromosome organization” and “microtubule-based process”, in accordance with the previous observations that cellular differentiation in the germline is associated with cell-specific changes in chromatin organization, cell-cycle regulation, and cell wall composition^19,48–50^.

We also reconstructed the developmental trajectory of female germline-associated cell clusters. This provided a refined view of the gene expression dynamics occurring during MMC specification. We identified distinct expression patterns of DEGs along the pseudotime trajectory, which provided insight into the molecular control of MMC differentiation. The top 100 DEGs along the developmental trajectory included the MMC marker genes *DMC1* and *ASY1*, known to play essential roles in MMC development^25,28^. Cells in the female germline-associated clusters bifurcated into two branches of the developmental trajectory. By grouping the DEGs associated with the branching according to their branch-specific expression patterns, we identified candidates driving MMC differentiation or cell-type identity. For example, genes that displayed increased expression along the GB are largely involved in chromosome organization, cell cycle, cell wall organization, and posttranscriptional regulation of gene expression, consistent with previous reports of their critical roles in germline differentiation^25,29,32,35,49,51–54^. Notably, some genes exhibited a sharp increase near the branchpoint followed by a decrease along the GB and thus may be implicated in the initiation of germline cell-fate specification. These genes are predominantly involved in response to cytokinin and peptide biosynthetic processes, highlighting the importance of phytohormone- and peptide-mediated signal transduction in germline cell-fate determination^34,47,55–58^. In agreement with this notion, we found that the *ER* family genes express preferentially in the epidermis of distal nucellus and the integuments during megasporogenesis and play an important role in restricting MMC specification to a single cell and promoting MMC differentiation (Fig. 9).

The research of stem cell development usually mitigates the influence of cell-cycle genes^17^. Here, we study the specification of female germline cells, which is different from stem cells. Previous studies have shown that the cell-cycle regulators such as ICK/KRPs and E2Fs play important roles in female germline specification^25,32^. In this study, we found that cell-cycle genes displayed increased expression along the germline growth. Their expression pattern is similar to the MMC marker genes (Fig. 4e and Supplementary Data 11), suggesting that they may also be involved in female germline differentiation. We did not mitigate cell-cycle gene effects before cell clustering to avoid leaving out the potential cell-cycle gene participation in germline specification.

In summary, our single-cell transcriptional atlas of *Arabidopsis* ovule primordia at three developmental time points during MMC specification provides details of ovule development at high resolution and in an unsupervised manner. From this dataset, we identified the female germline lineage as well as the surrounding soma sub-populations. This study reveals the cellular heterogeneity in ovule primordia, reconstructs the continuous developmental trajectory of the female plant germline, and illustrates the gene expression dynamics during MMC differentiation. Further, we established a set of TF connection networks coordinated with the germline cell differentiation trajectory. Integrated analysis of the three samples consistently identified the feature genes of the female germline cell clusters and revealed the transcriptional dynamics during MMC development. We anticipate that the valuable resource provided by these data will enable a better understanding of the molecular control of female germline differentiation, a key process in sexual plant reproduction.

## Methods

### Plant materials and growth conditions

The homozygous MMC marker line *pKNU::KNU-Venus* in the *Landsberg erecta* (*Ler*) background was used for single-cell RNA-seq. *er-105*, *erl1-2* and *erl2-1*, complete loss-of-function alleles of *ER* (AT2G26330), *ERL1* (AT5G62230), and *ERL2* (AT5G07180), were used to construct *er-105 erl1-2 erl2-1* mutant^59^. Plants were grown in soil under 16 h light/8 h dark at 22 °C.

### Protoplast isolation

Protoplasts were isolated as described with minor modifications^4^. Protoplast digestion mixture was prepared fresh by adding 1.5% Cellulase R-10 and 0.2% Macerozyme R-10 (Yakult Company) to fresh protoplast buffer (1 M KCl, 1 M MgCl_2_, 1 M CaCl_2_, 0.1% BSA (Sigma Aldrich), 0.04% MES, and 1% mannitol) and mixed thoroughly. We monitored the morphology of floral organs to judge the developmental stage of the flower and ovule^24,60–62^. Briefly, for each sample, ovule primordia with placenta dissected from 60 *pKNU::KNU-Venus* floral buds at floral stages 9–10 were added to 2 ml of protoplast digestion mixture for digestion and incubated for 3 h with mild shaking at 100 rpm. The digestion mixture with protoplasts was filtered through a 40-μm-pore-size cell strainer to remove a small amount of undigested tissue, and the filtrate was centrifuged at 500×*g* for 5 min at room temperature. The supernatant was gently removed and resuspended with 1 ml fresh protoplast buffer. Protoplasts were validated under a Countstar instrument (Countstar Rigel S2). The proportion of living cells, cell concentration, and group rate are provided in Supplementary Data 1. Finally, the suspension volume was adjusted to a density of 500–1000 cells/μl.

### Single-cell RNA-seq library construction and sequencing

Single-cell RNA-seq libraries of fresh protoplasts were generated according to the protocol for the 10X Genomics Single Cell 3’ Reagent Kit v2. The Unique alignment of RNA sequences and UMI (Unique Molecular Identifier) were collated to remove the duplicated PCR product to estimate the number of valid cells (Supplementary Fig. 2a–c and Supplementary Fig. 18b). For each sample, ~6000 valid cells were captured with the 10X Genomics Chromium single-cell microfluidics device. Twelve cycles were used for cDNA amplification, and final library size and quality were assessed on an Agilent Bioanalyzer High Sensitivity chip. Then, libraries were quantified using the NEBNext Library Quantification Kit for Illumina. Finally, scRNA-seq library sequencing was performed on the NextSeq (Illumina) platform using the default parameters (a sequencing depth of at least 100,000 reads per cell with pair-end 150 bp (PE150) reading strategy (performed by CapitalBio Technology, Beijing)). All the cells in three samples were integrated together by Cell Ranger AGGR pipeline and normalized by equalizing the read depth among libraries.

### Generation of single-cell expression matrices

The raw scRNA-seq data were analyzed and mapped to the TAIR10 *Arabidopsis* genome using the Cell Ranger pipeline 2.1.0 (10X Genomics). The Cell Ranger pipeline was used to count the number of cells and mean reads per cell by cell barcode and UMI (unique molecule identifiers), and to produce digital expression matrices for each sample.

### Principal component analysis (PCA), t-SNE visualization, cell clustering, and correlation analysis

PCA and *t*-distributed stochastic neighbor-embedding (t-SNE) and uniform manifold approximation and projection (UMAP) were used for dimensionality reduction and visualization analysis with the prcomp and Rtsne packages of the R software (Version 3.4.1). Variable genes were identified across the single cells, using the relationship between average expression and dispersion. PCA was performed using the variable genes as input, and the number of principal components was determined from a screen plot. t-SNE was then performed on the first ten principal components with default parameters to visualize cells and for clustering analysis in a two-dimensional space. The Pearson correlation coefficient was used to calculate correlation analysis of clusters and subclusters in three samples. The average expression of genes in each cluster was used to analyze the correlation of clusters and subgroups.

### Identification of cluster identities

The gene-cell matrices of valid cells were load into the Loupe Cell Browser (version 3.1.0) to perform clustering, based on graph-based clustering. The transcript accumulation and gene expression levels of reported marker genes were used to determine cluster identities. Loupe Cell Browser was used to calculate the transcript accumulation of some marker genes in clusters and subclusters using the log2 fold change (log2FC) with Feature Max. In a complementary approach, marker genes for key cell types were identified from specific clusters or subclusters. Cells expressing marker genes (log2FC > 0) were filtered from each cluster and the percentage of specific cells in the cluster was calculated. The expression profiles of genes involved in the regulation of MMC in clusters and subclusters were plotted using the R package (pheatmap and monocle2).

### Heatmap analysis of gene expression

Characteristic genes in each cluster were obtained by comparing the differentially expressed genes (DEGs) (log2FC > 1, *P* < 0.05) in each cluster among other clusters in the same sample. Characteristic genes met the criteria of mean UMI counts >1.0, and then the top 20 genes were selected as candidate characteristic genes for this cell population by ranking with the log2FC value.

Monocle finds DEGs (Scale[log2(count)] >1, *P* < 0.05) with the differentialGeneTest function. The heat map describing waves of gene expression across pseudotime was obtained by comparing gene expression of cells in differential states using differentialGeneTest.

### Gene ontology (GO) enrichment analysis

GO biological process enrichment analysis was carried out using AgriGO (http://bioinfo.cau.edu.cn/agriGO/). The biological processes with the differentially expressed genes of each cluster in the three samples and cluster-grouped differentially expressed genes along pseudotime were used for heatmap analysis in R (*P* < 0.05).

### Single-cell developmental trajectory analysis

To explore the single-cell developmental trajectory, the Monocle 2 R package (version 2.8.0) was used to analyze the pseudotime trajectory of cell differentiation and the determination of cell fate. The analysis was carried out based on the average expression levels of variable genes in the targeted cells (gene expression in cell >10, average expression >0.5, and *P* < 0.01), the cell trajectory was plotted and visualized in a reduced two components. The “beginning” of the trajectory was specified by “orderCells”, and pseudotime trajectory analysis was performed using the Monocle 2 R package (version 2.8.0) algorithm^36^. Differentially expressed genes along the pseudotime and the default branchpoint 1 were identified using the differentialGeneTest function (*q* < 0.01). Genes dynamically expressed along the pseudotime were clustered using the ‘plot_pseudotime_heatmap’ function with the default parameters. The genes with branch-dependent expression were visualized by the “plot_genes_branched_ heatmap” function with default parameters.

### Gene-regulatory network analysis

The TFs differentially expressed at branchpoint 1 were annotated in PlantTFDB (http://planttfdb.cbi.pku.edu.cn/). Gene-regulatory network inference was calculated on the TFs using String (https://string-db.org/). Gene-regulatory inference was filtered using different cutoffs on the parameter value. The gene correlation networks were visualized using Cytoscape, and the network topological parameters were obtained with NetworkAnalyzer^63^.

### Vector construction and transformation

To generate the *pER:GFP*/*pERL1:GFP*/*pERL2:GFP*/*pPGDH1:3×Venus*/*pAT4G13710: 3×Venus* construct, the promoter sequences of *ER* (AT2G26330), *ERL1* (AT5G62230), *ERL2* (AT5G07180) *pPGDH1* (AT4G34200), and *AT4G13710* were amplified, and PCR fragments were cloned into the pENTR/D-TOPO vector (Invitrogen) and the pPGDH1/AT4G13710 3×Venus -N7 vectors. The pENTR clones were recombined into the destination vector pGWB604 using LR Clonase II (Invitrogen). The resulting construct also contained the selectable marker BAR for glufosinate resistance^64^.

WT *Arabidopsis* (Col-0) plants were then infected with the transformed bacteria by the floral dip method^65^. All the primers used in the article are listed in Supplementary Data 14.

### Confocal microscopy of ovules

Ovules collected from pistils at different stages of flower development were incubated in FM4-64 for 5 min and then analyzed using Leica SP8 microscope at excitation wavelengths of 488 nm. For the expression pattern analysis of the transgenic lines, more than three independent transgenic lines were observed and confirmed to have similar patterns.

### Resin semi-thin sectioning

The inflorescences of the MMC marker line *pKNU::KNU-Venus* were fixed overnight or longer in FAA (38% formalin: acetic acid: 70% alcohol=2:1:10), dehydrated by gradient series of ethanol (30%, 50%, 70%, 80%, 90%, 95%, and 100%), then infiltrated with Eponate 812 resin, followed by embedding and polymerization. Semi-thin sections (1 μm) were generated with a Leica (RM2255) microtome and stained with 0.1% toluidine blue. Images were obtained with an Olympus microscope (BX63).

### DIC observation of ovule structure

The inflorescences from 6 to 8 wild-type and *er erl1 erl2* plants were fixed in FAA overnight or longer. The pistils of the flowers at 9–10 stages were dissected in a drop of chloral hydrate solution (chloral hydrate: H_2_O: glycerol = 8:2:1). Images of ovules were obtained with a BX63 microscope (Olympus) using a ×40 objective.

### Whole-mount immunolocalization with ovules

Ovules with placenta from pistils of stages 9–10 flowers were dissected, fixed, and processed as described in the published protocol^45^. The AGO9 primary antibody (Agrisera, AS10673) was used at a dilution of 1:100. The secondary antibody Alexa Fluor 488 (Molecular Probes) was used at a dilution of 1:500. Before mounting, the samples were incubated with propidium iodide (PI) (500 mg/mL). A confocal microscope (Leica TCS SP5) was used to capture images. For PI detection, excitation was at 568 nm and emission at 575–615 nm. For Alexa Fluor 488, excitation and emission were at 488 and 500–550 nm, respectively. The laser intensity and gain of all experiments were set at similar levels.

### Statistics and reproducibility

The detailed information on the experimental design and statistics used in the different data analyses performed in this study are given in the various sections of Results and Methods. DEGs (Scale[log2(count)] >1, *P* < 0.05) were identified by Monocle with the differentialGeneTest function. GO biological process enrichment analysis was carried out using AgriGO (http://bioinfo.cau.edu.cn/agriGO/). For the analysis of the expression of *pKNU:KNU-Venus* in the ovule primordia and the germline specification phenotype of *er-105 erl1-2 erl2-1* triple mutant, we performed three independent biological repetitions and each repetition included three floral buds. The statistical significance of the difference between wild-type and mutants was determined using Student’s *t* test.

### Reporting summary

Further information on research design is available in the Nature Research Reporting Summary linked to this article.

## Supplementary information

Supplementary Information

Description of Additional Supplementary Files

Supplementary Data 1.

Supplementary Data 2.

Supplementary Data 3.

Supplementary Data 4.

Supplementary Data 5.

Supplementary Data 6.

Supplementary Data 7.

Supplementary Data 8.

Supplementary Data 9.

Supplementary Data 10.

Supplementary Data 11.

Supplementary Data 12.

Supplementary Data 13.

Supplementary Data 14.

Reporting Summary

## Data Availability

scRNA sequencing raw data used in this study have been deposited at the European Nucleotide Archive (ENA) and is accessible via accession number PRJEB47244. The data supporting the findings of this study are available within Supplementary Data. And all other data are available from the corresponding author (or other sources, as applicable) on reasonable request.

## Code availability

Details of the public software used in the study can be found in “Methods”. No custom code or mathematical algorithms that is deemed central to the conclusions was used.
