# Supplementary material for: High-throughput single-cell transcriptomics reveals the female germline differentiation trajectory in Arabidopsis thaliana
Source: Commun Biol. 2021 Oct 1;4:1149. doi: 10.1038/s42003-021-02676-z (PMC8486858; doi:10.1038/s42003-021-02676-z)
Supplement: Supplementary file 1 — Supplementary Information [file 42003_2021_2676_MOESM1_ESM.pdf]

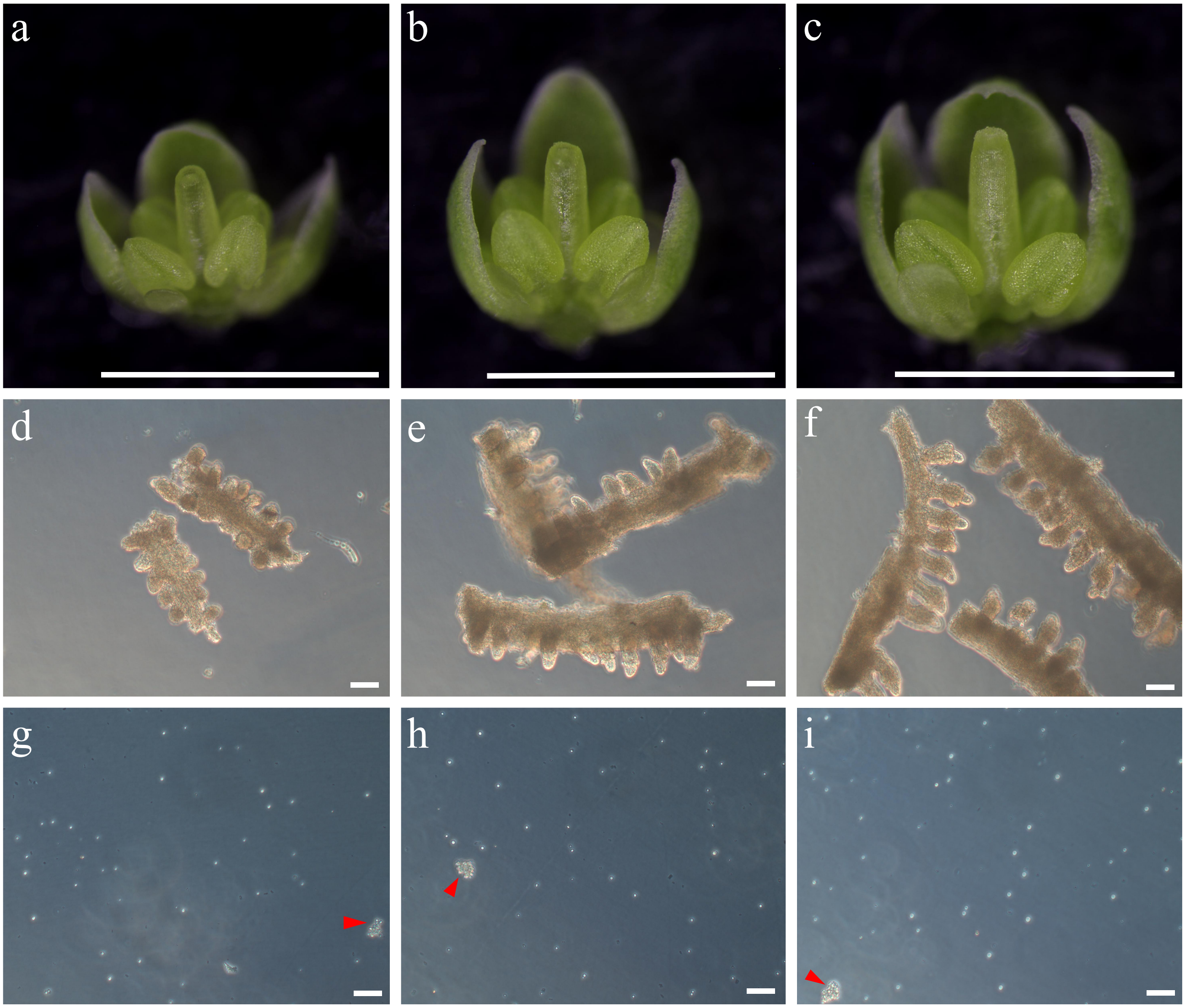

Supplementary Fig. 1. The morphology of floral buds and the AC, MMC1 and MMC2 stages of ovule samples before and after enzymolysis.

a–c, The morphology of floral buds used to dissect the ovule samples at AC (**a**), MMC1 (**b**) and MMC2 (**c**) stages. Scale bars, 1mm.

d-f, The ovule primordia samples with placenta at AC (**d**), MMC1 (**e**) and MMC2 (**f**) stages dissected from the floral buds. Scale bars, 50 µm.

g-i, Protoplast suspension of the ovule primordia samples at AC (**g**), MMC1 (**h**) and MMC2 stages (**i**), Scale bars, 50 µm. Red triangles indicate tissue pieces remained after enzymolysis.

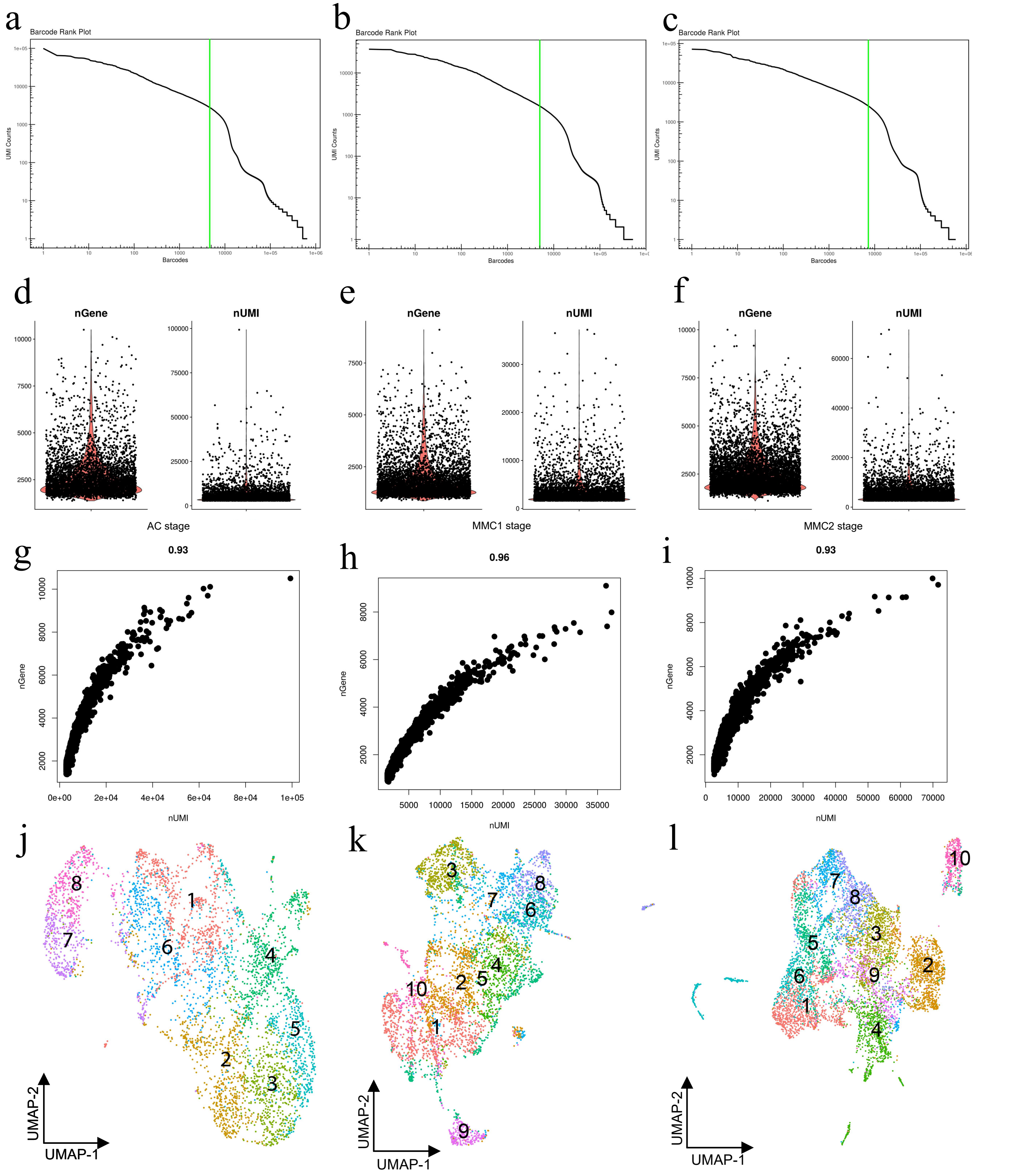

Supplementary Fig. 2. Evaluation of effective cell, genes and transcripts (UMIs) detected per cell in ovule samples at AC, MMC1 and MMC2 stages.

a-c, Evaluation of effective cell in the ovule samples at AC (**a**), MMC1 (**b**) MMC2 (**c**) stages. Horizontal axis: number of barcodes; vertical axis: counts of unique molecular identifiers (UMIs). On the X-axis, barcode on the left side of the green marker line is used as the active cell, while barcode on the right side is used as the background.

d–f, Violin plots of genes (left) and transcripts (UMIs; right) in the ovule samples at AC (**d**), MMC1 (**e**) and MMC2 (**f**). The sample names are shown on the abscissa and the number of genes or UMIs in each cell is shown on the ordinate. Violin Plot is used to display the distribution status and probability density of multiple sets of data.

g–i, Correlation between gene number and transcript (UMI) number in the AC (**g**), MMC1 (**h**) and MMC2 (**i**) stages of ovule samples. The UMI number of each cell is on the abscissa and the gene number is on the ordinate.

j-l, Visualization of cell clusters for the ovule samples at AC (**j**), MMC1 (**k**) and MMC2 (**l**) stages using UMAP. Dots, individual cells; color, cell clusters.

AC

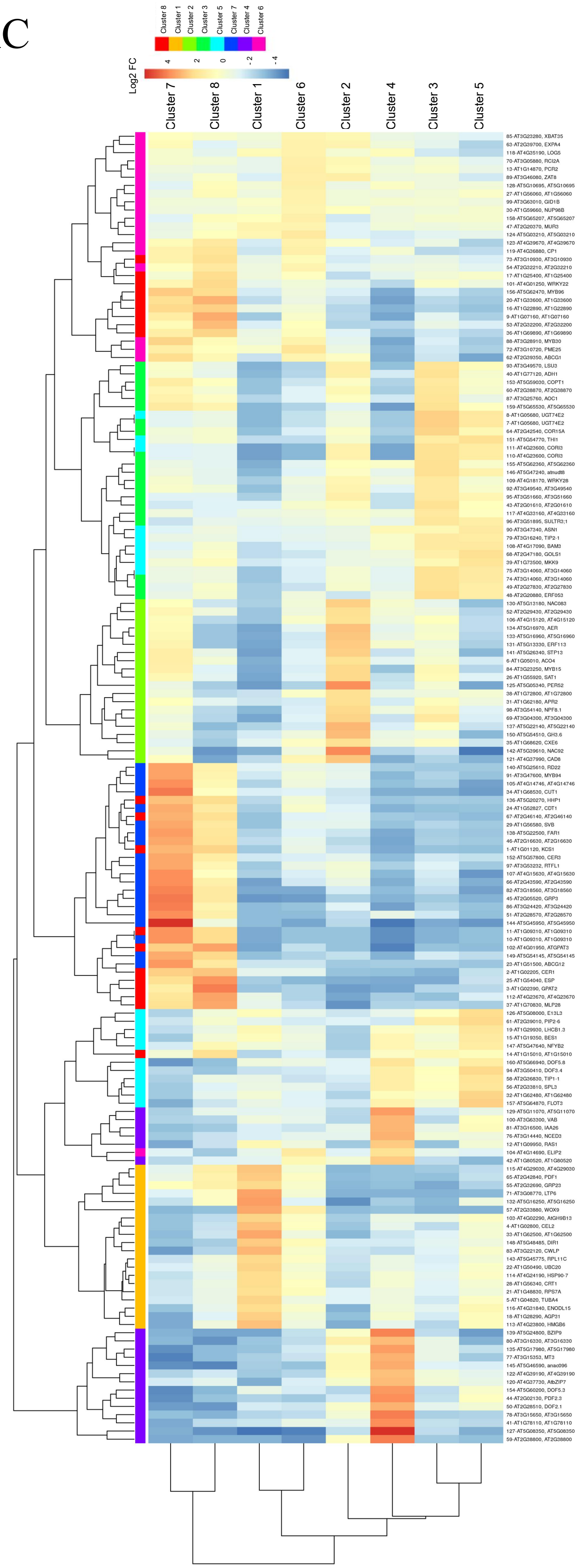

Supplementary Fig. 3. Heatmap displaying the expression of the top 20 feature genes of each cell cluster in ovule samples from the AC stage across clusters. Each column represents the cell cluster and each row represents one gene. The rainbow color bar indicates cell cluster identity. The red-to-blue color bar represents the average gene expression level (Log2 FC) from high to low.

MMC1

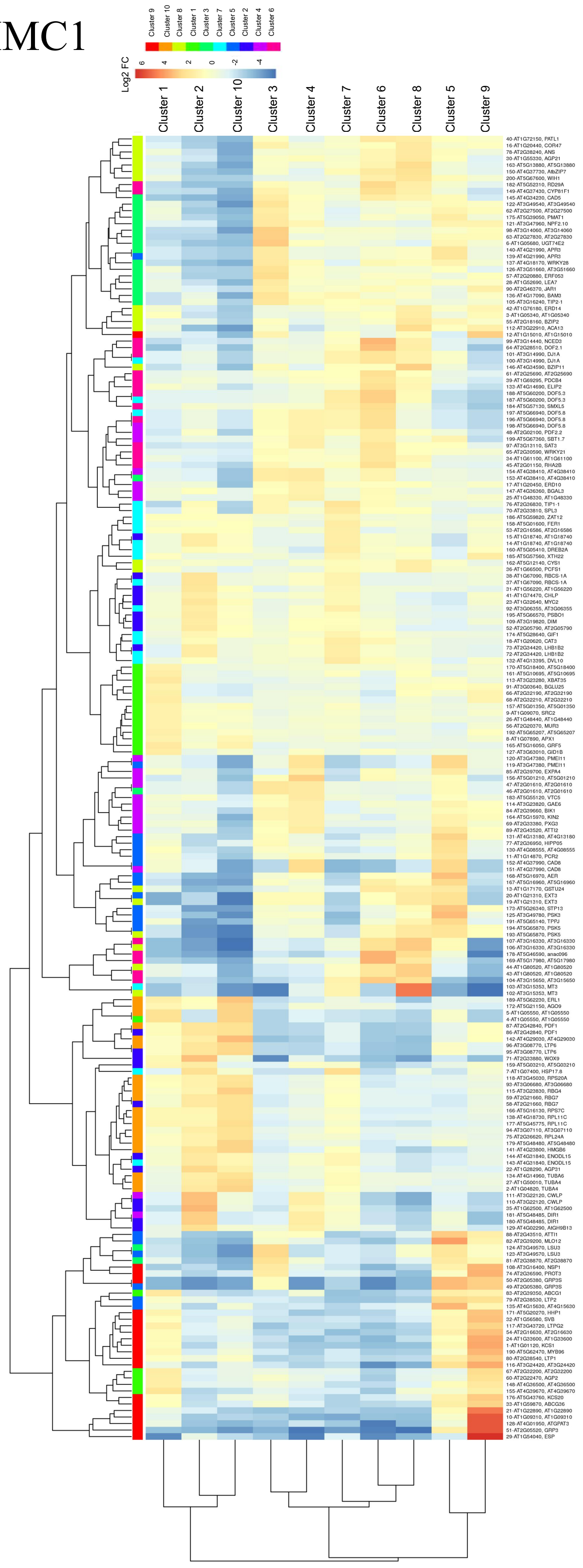

Supplementary Fig. 4. Heatmap displaying the expression of the top 20 feature genes of each cell cluster in ovule samples from the MMC1 stage across clusters. Each column represents the cell cluster and each row represents one gene. The rainbow color bar indicates cell cluster identity. The red-to-blue color bar represents the average gene expression level (Log2 FC) from high to low.

MMC2

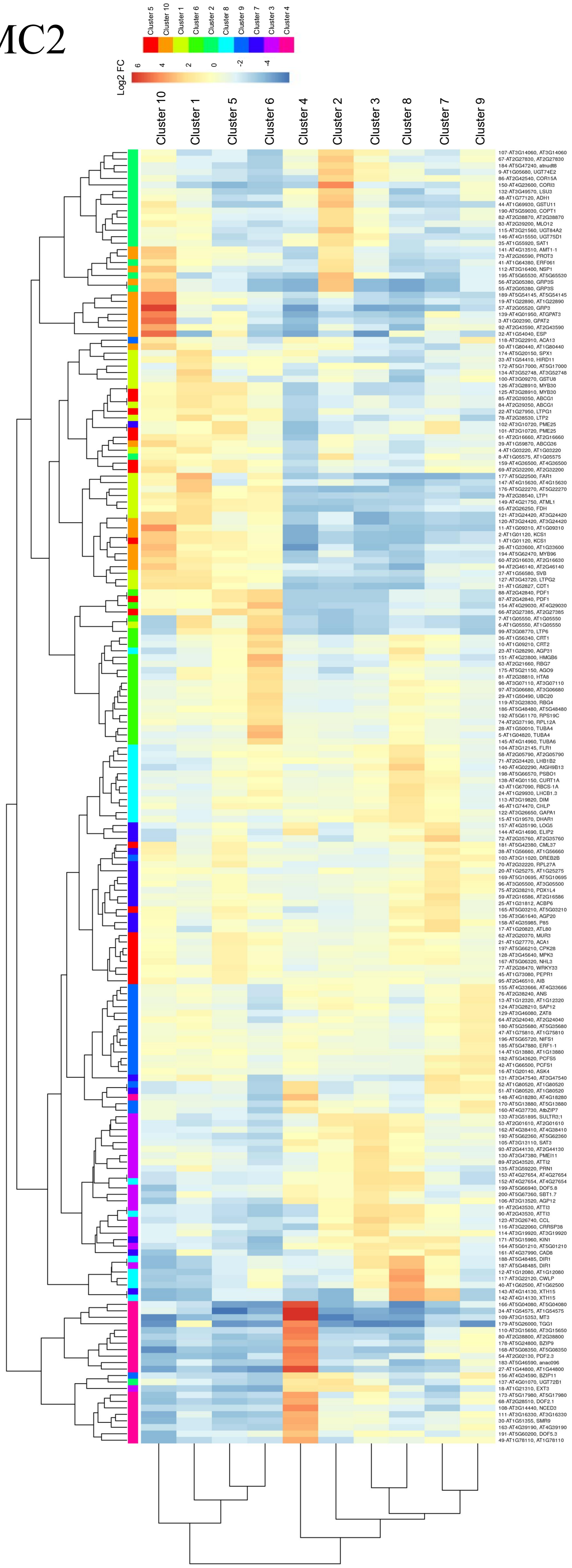

Supplementary Fig. 5. Heatmap displaying the expression of the top 20 feature genes of each cell cluster in ovule samples from the MMC2 stage across clusters. Each column represents the cell cluster and each row represents one gene. The rainbow color bar indicates cell cluster identity. The red-to-blue color bar represents the average gene expression level (Log2 FC) from high to low.

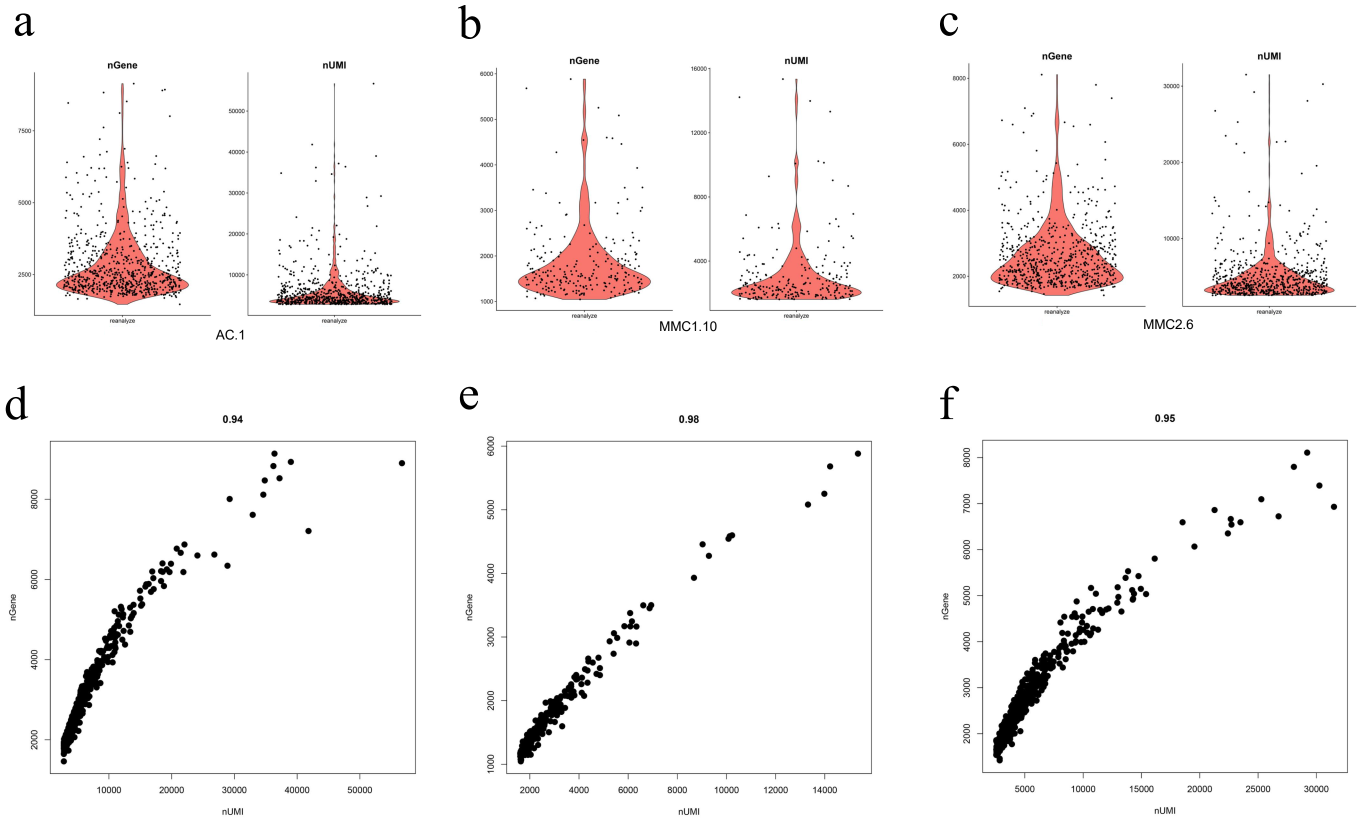

Supplementary Fig. 6. Genes and transcripts (UMIs) detected per cell in the AC.1, MMC1.10 and MMC2.6 cell clusters.

a–c, Violin plots of genes (left) and transcripts (unique molecular identifiers, UMIs; right) in the AC.1 **(a)**, MMC1.10 **(b)** and MMC2.6 **(c)** cell clusters. The sample names are shown on the abscissa and the number of genes or UMIs in each cell is shown on the ordinate. Violin Plot is used to display the distribution status and probability density of multiple sets of data.

d–f, Correlation between gene number and transcript (UMI) number in the AC.1 **(d)**, MMC1.10 **(e)** and MMC2.6 **(f)** cell clusters. The UMI number of each cell is on the abscissa and the gene number is on the ordinate.

AC.1

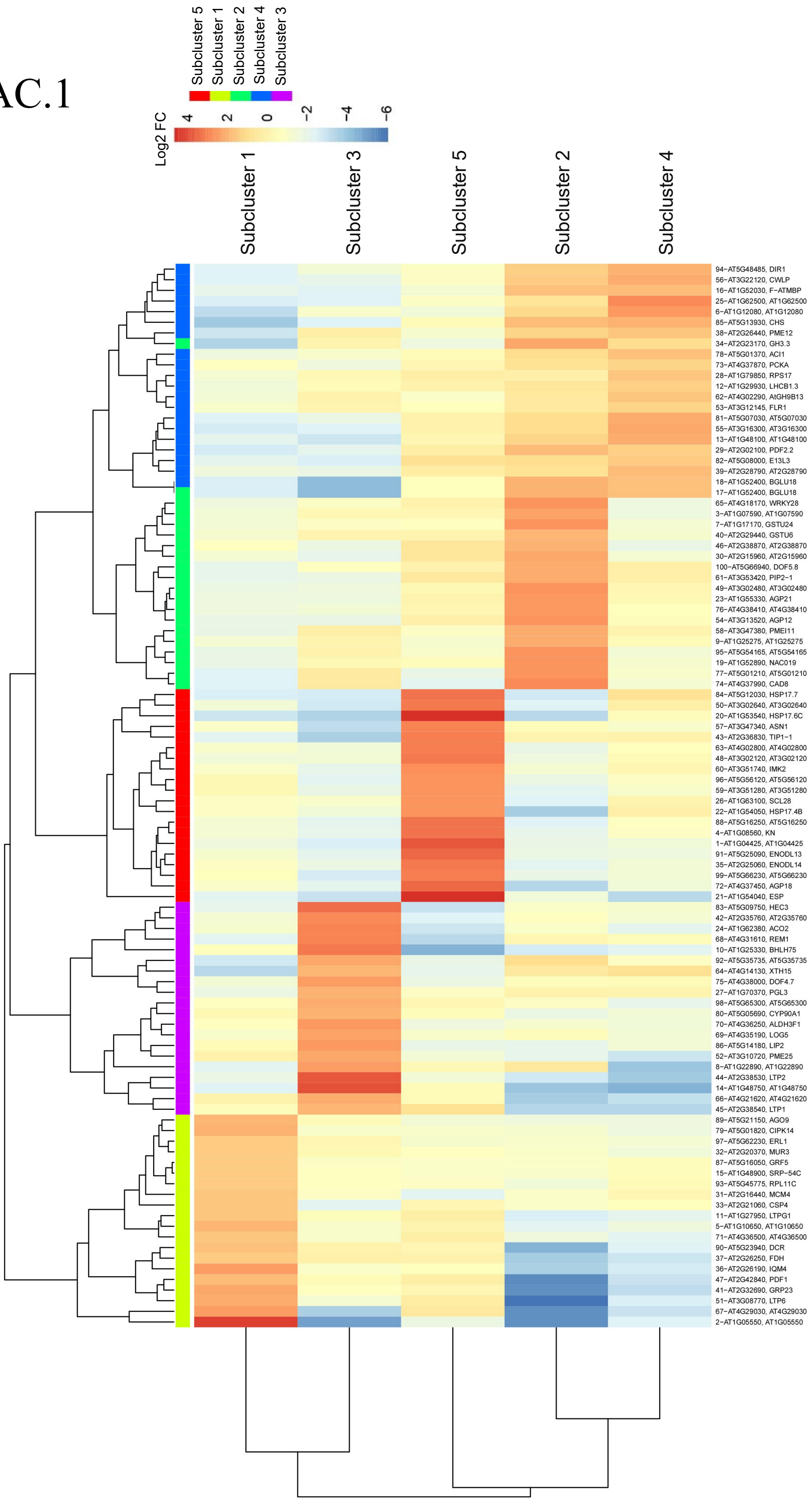

Supplementary Fig. 7. Heatmap displaying the expression of the top 20 feature genes of each cell cluster in the AC.1 cluster across the subclusters. Each column represents the cell cluster and each row represents one gene. The rainbow color bar indicates cell cluster identity. The red-to-blue color bar represents the average gene expression level (Log2 FC) from high to low.

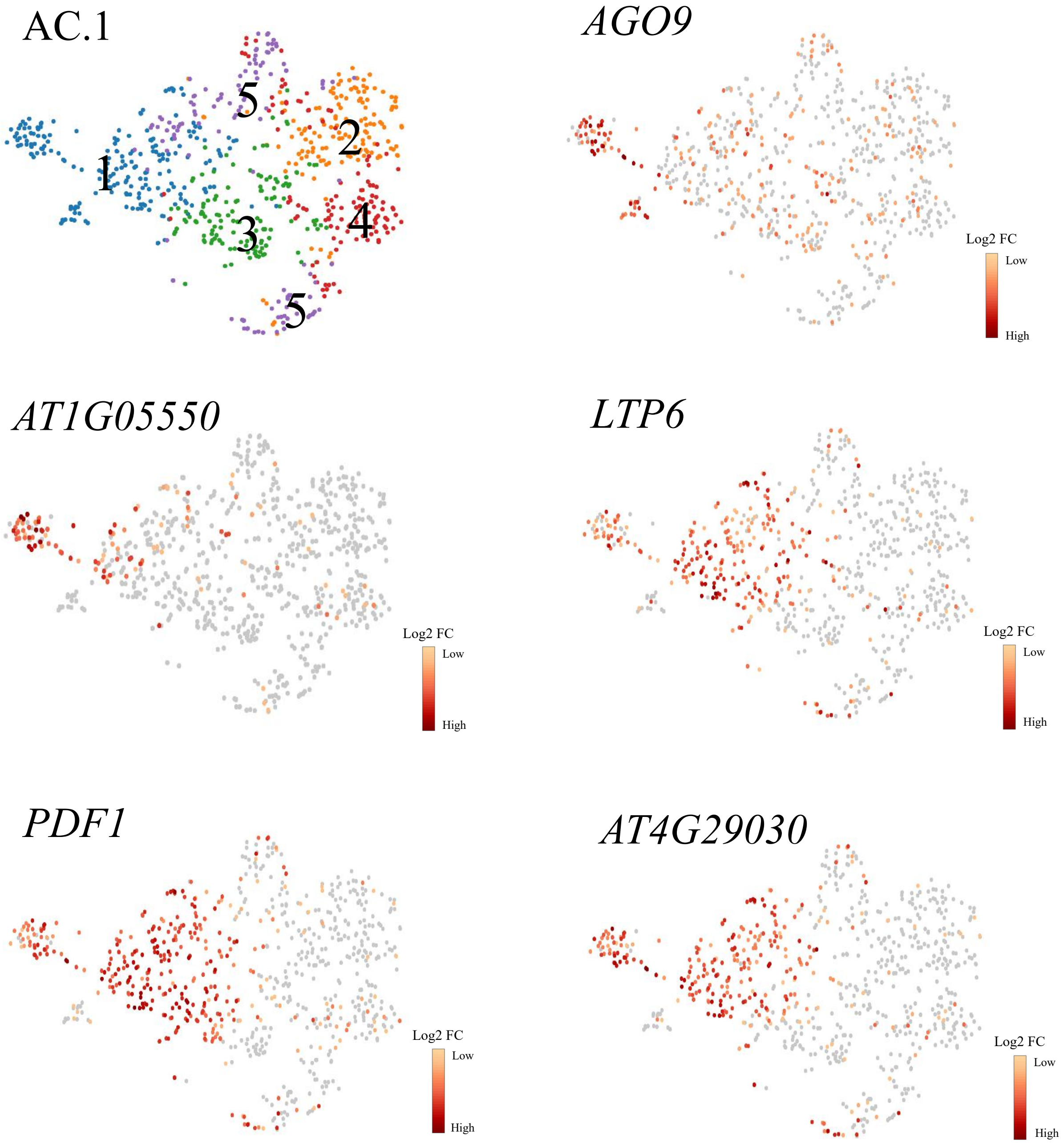

Supplementary Fig. 8. t-SNE visualization indicating the transcript accumulation for the feature genes (*AOG9*, *AT1G05550*, *LTP6*, *PDF1* and *AT4G29030*) of the female germline sub-cluster in individual cells of the AC.1 cell cluster. Color intensity indicates the relative transcript level (Log2 FC) for the indicated gene in each cell.

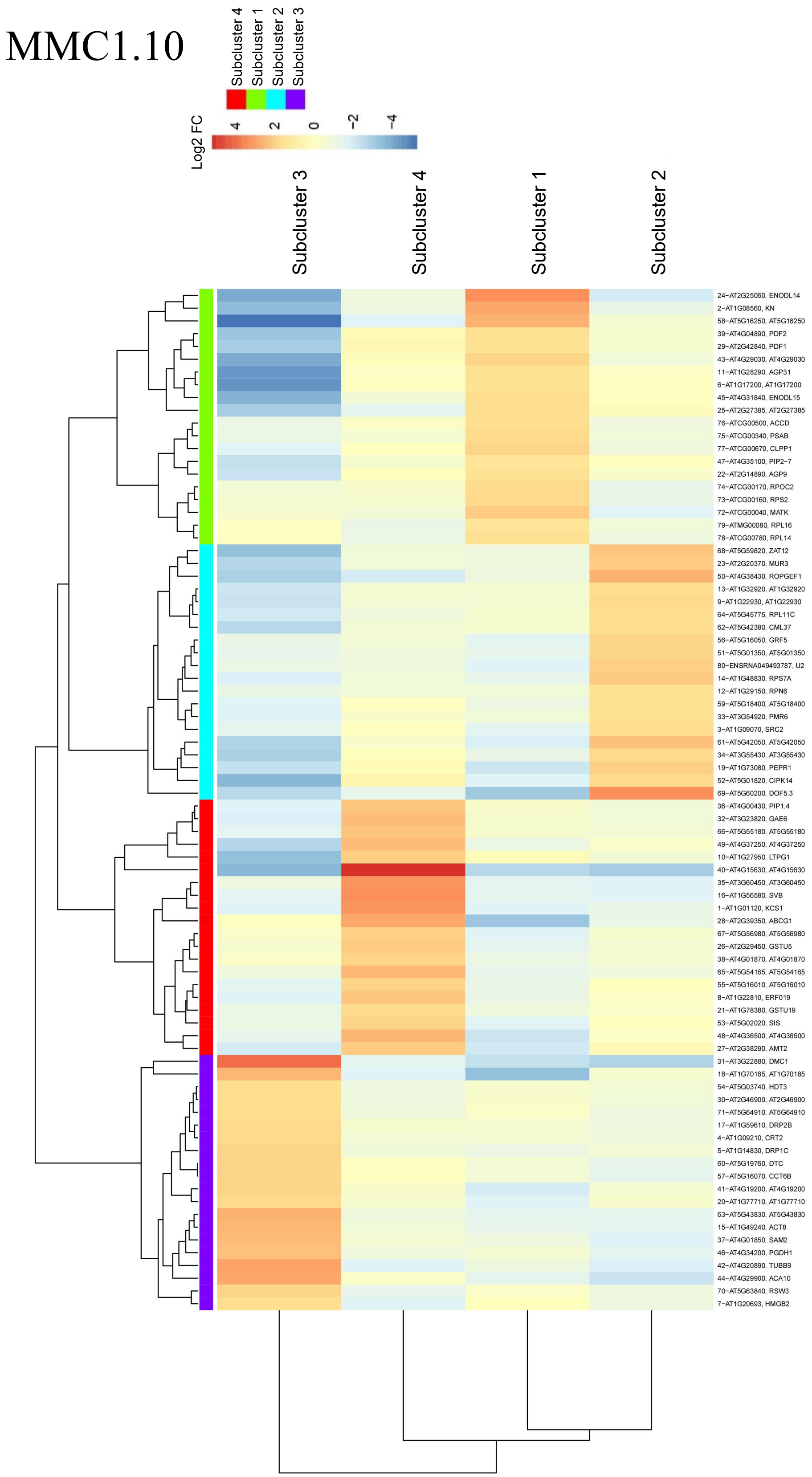

Supplementary Fig. 9. Heatmap displaying the expression of the top 20 feature genes of each cell cluster in the MMC1.10 cluster across the subclusters. Each column represents the cell cluster and each row represents one gene. The rainbow color bar indicates cell cluster identity. The red-to-blue color bar represents the average gene expression level (Log2 FC) from high to low.

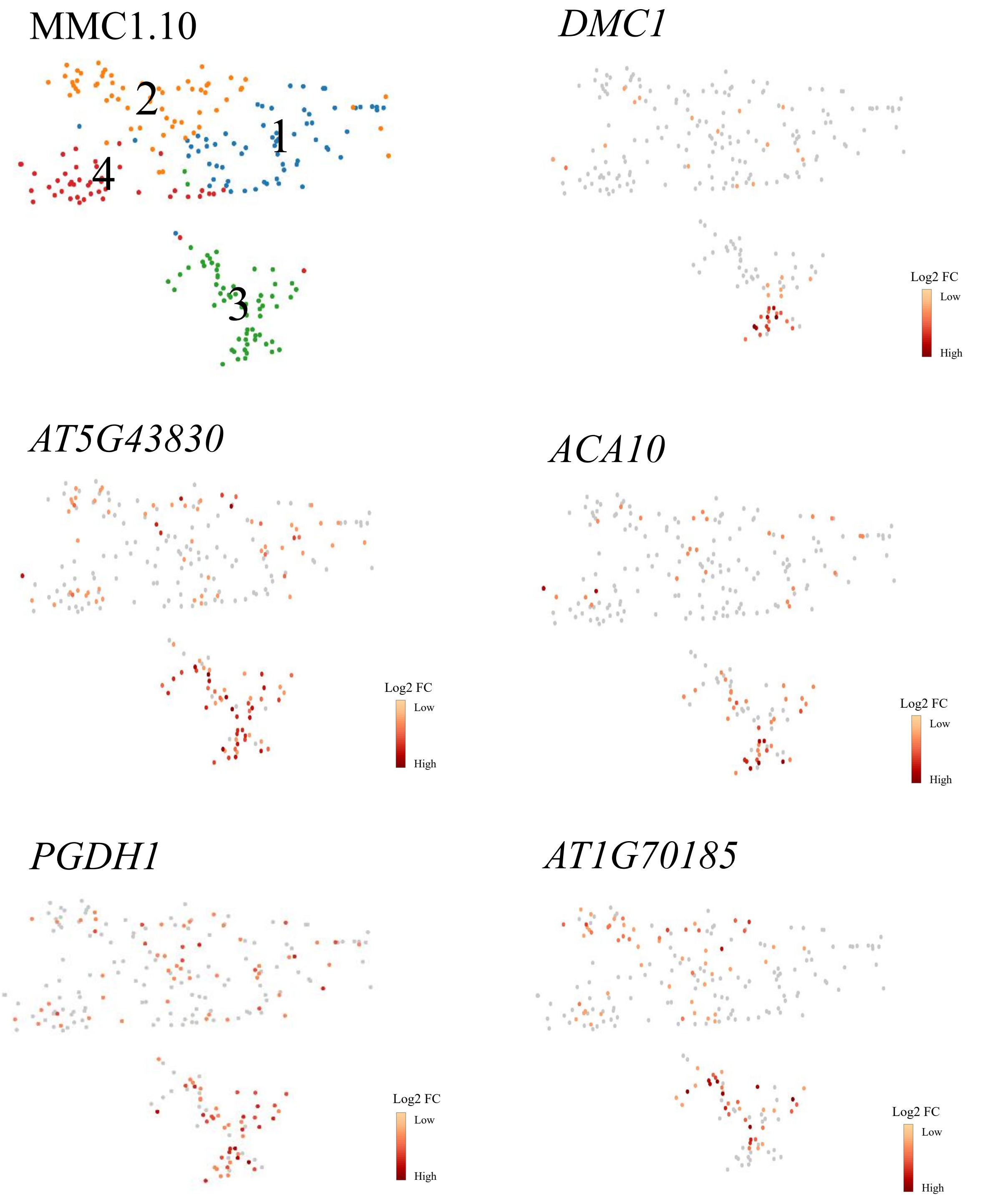

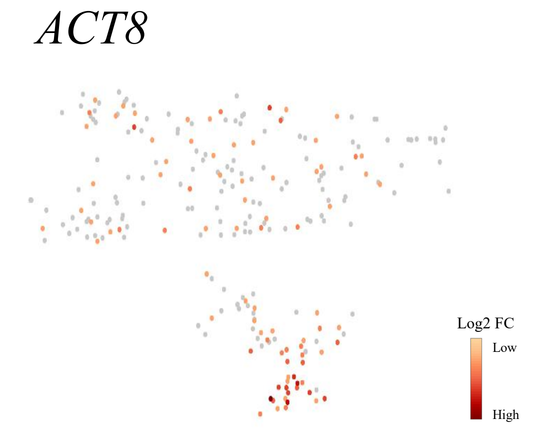

Supplementary Fig. 10. t-SNE visualization indicating the transcript accumulation for the feature genes (*DMC1*, *AT5G43830*, *ACA10*, *PGDH1*, *AT1G70185* and *ACT8*) of the female germline sub-cluster in individual cells of the MMC1.10 cell cluster. Color intensity indicates the relative transcript level (Log2 FC) for the indicated gene in each cell.

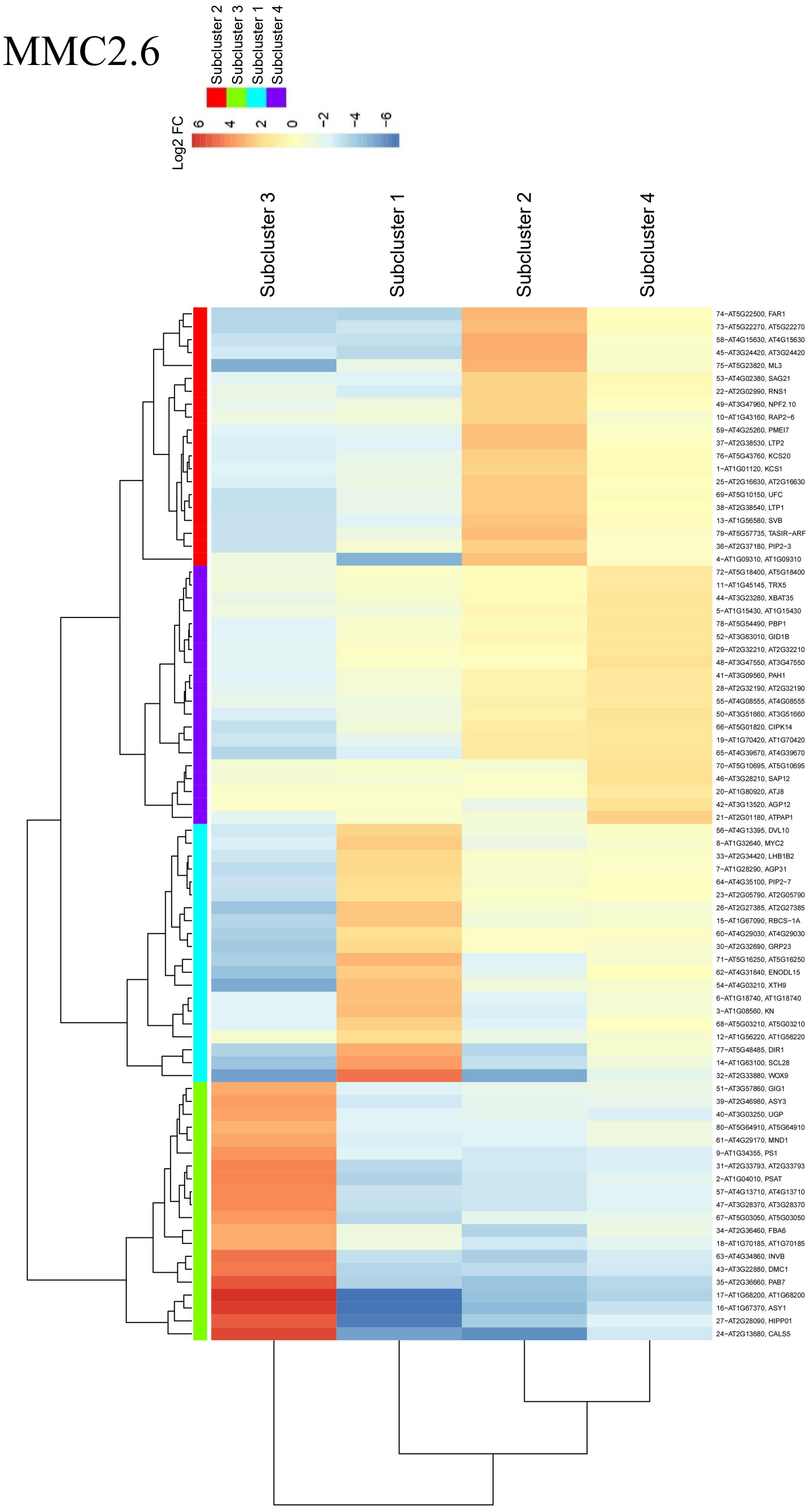

Supplementary Fig. 11. Heatmap displaying the expression of the top 20 feature genes of each cell cluster in the MMC2.6 cluster across the subclusters. Each column represents the cell cluster and each row represents one gene. The rainbow color bar indicates cell cluster identity. The red-to-blue color bar represents the average gene expression level (Log2 FC) from high to low.

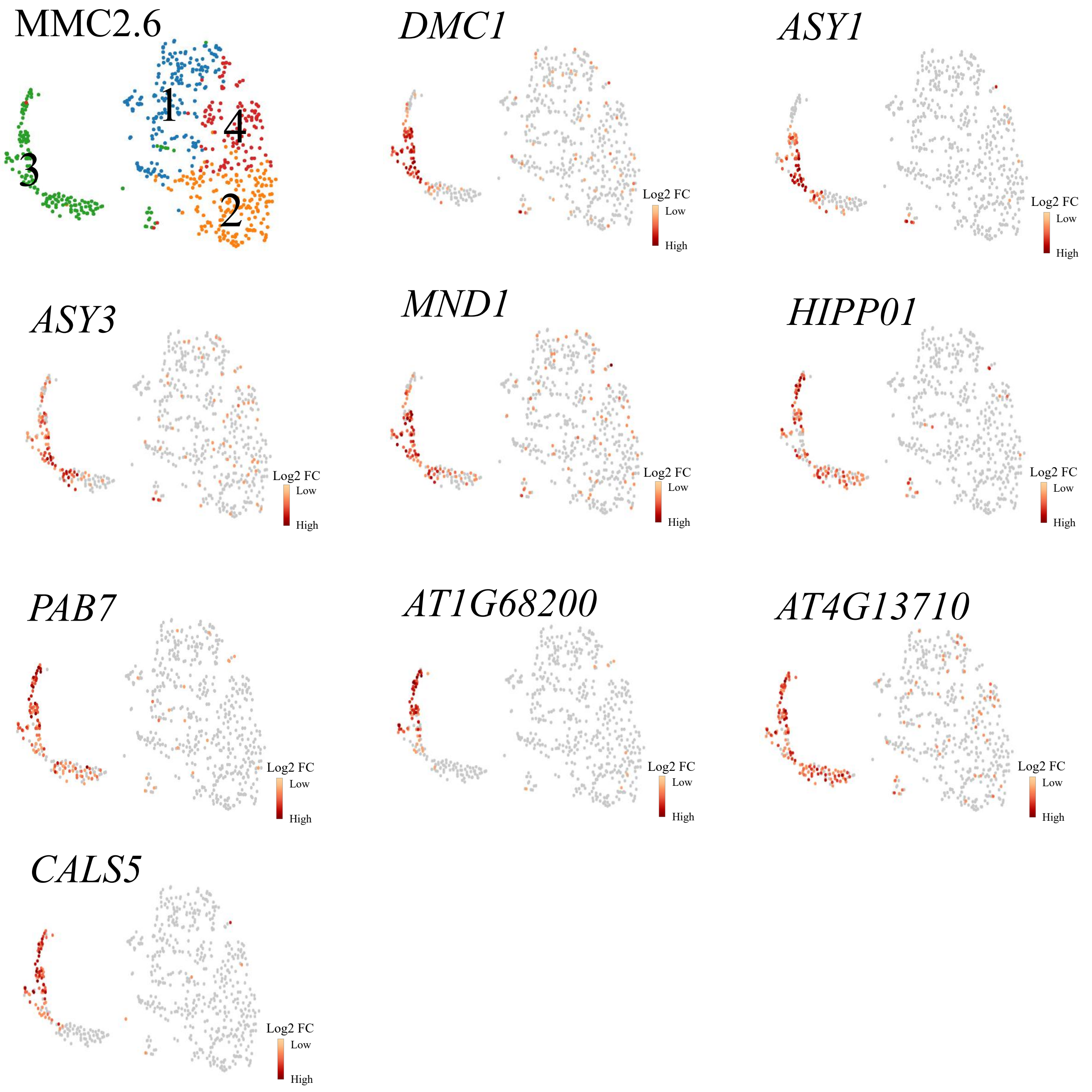

Supplementary Fig. 12. t-SNE visualization indicating the transcript accumulation for the feature genes (*DMC1*, *ASY1*, *ASY3*, *MND1*, *HIPP01*, *PAB7*, *AT1G68200*, *AT4G13710* and *CALS5*) of the female germline sub-cluster in individual cells of the MMC2.6 cell cluster. Color intensity indicates the relative transcript level (Log2 FC) for the indicated gene in each cell.

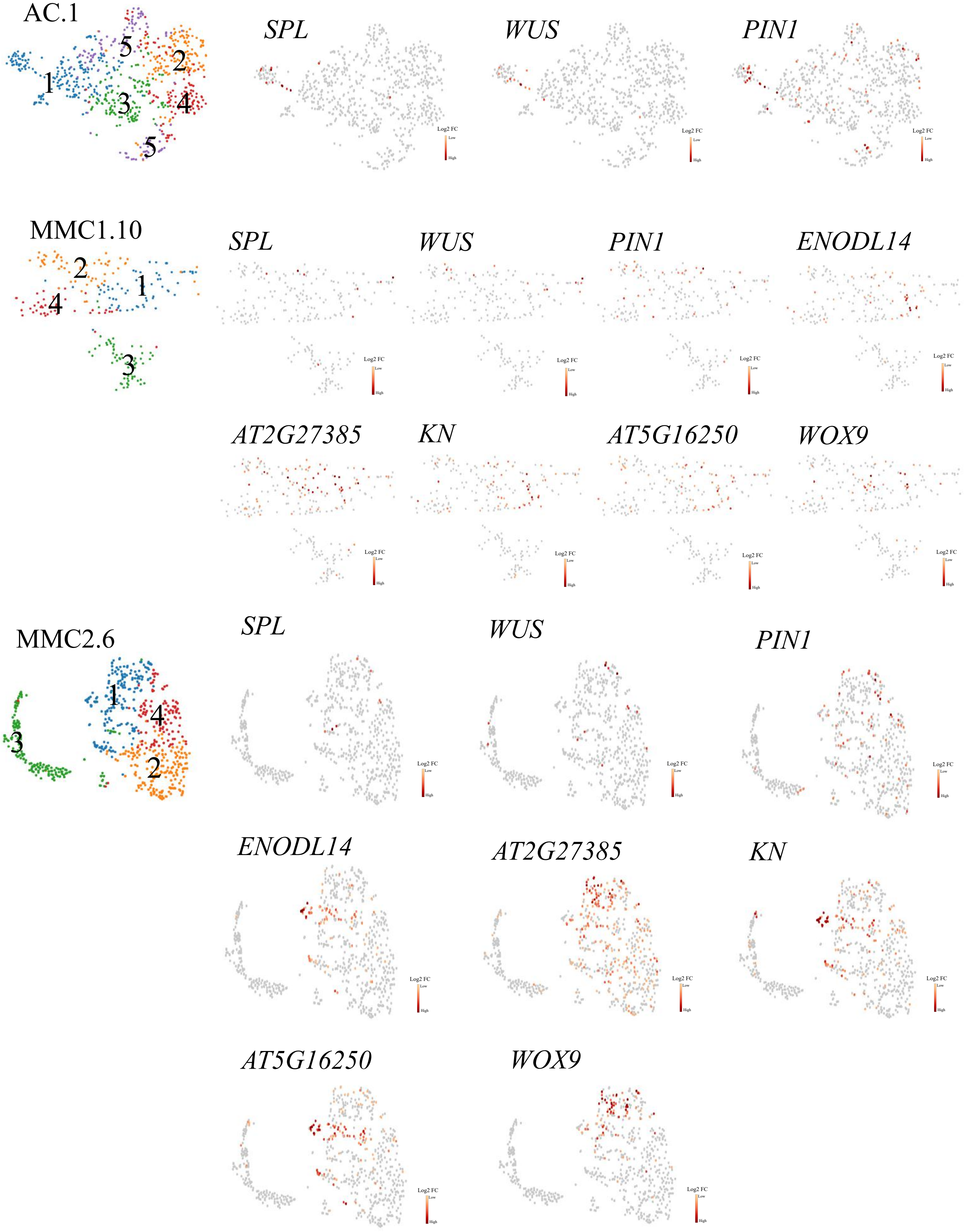

Supplementary Fig. 13. t-SNE visualization indicating the transcript accumulation for the marker genes (*SPL*, *WUS*, *PIN1*) and the feature genes (*ENODL14*, *AT2G27385*, *KN*, *AT5G16250* and *WOX9*) of the nucellar epidermis cell sub-clusters in individual cells of the AC.1, MMC1.10 and MMC2.6 cell clusters. Color intensity indicates the relative transcript level (Log2 FC) for the indicated gene in each cell.

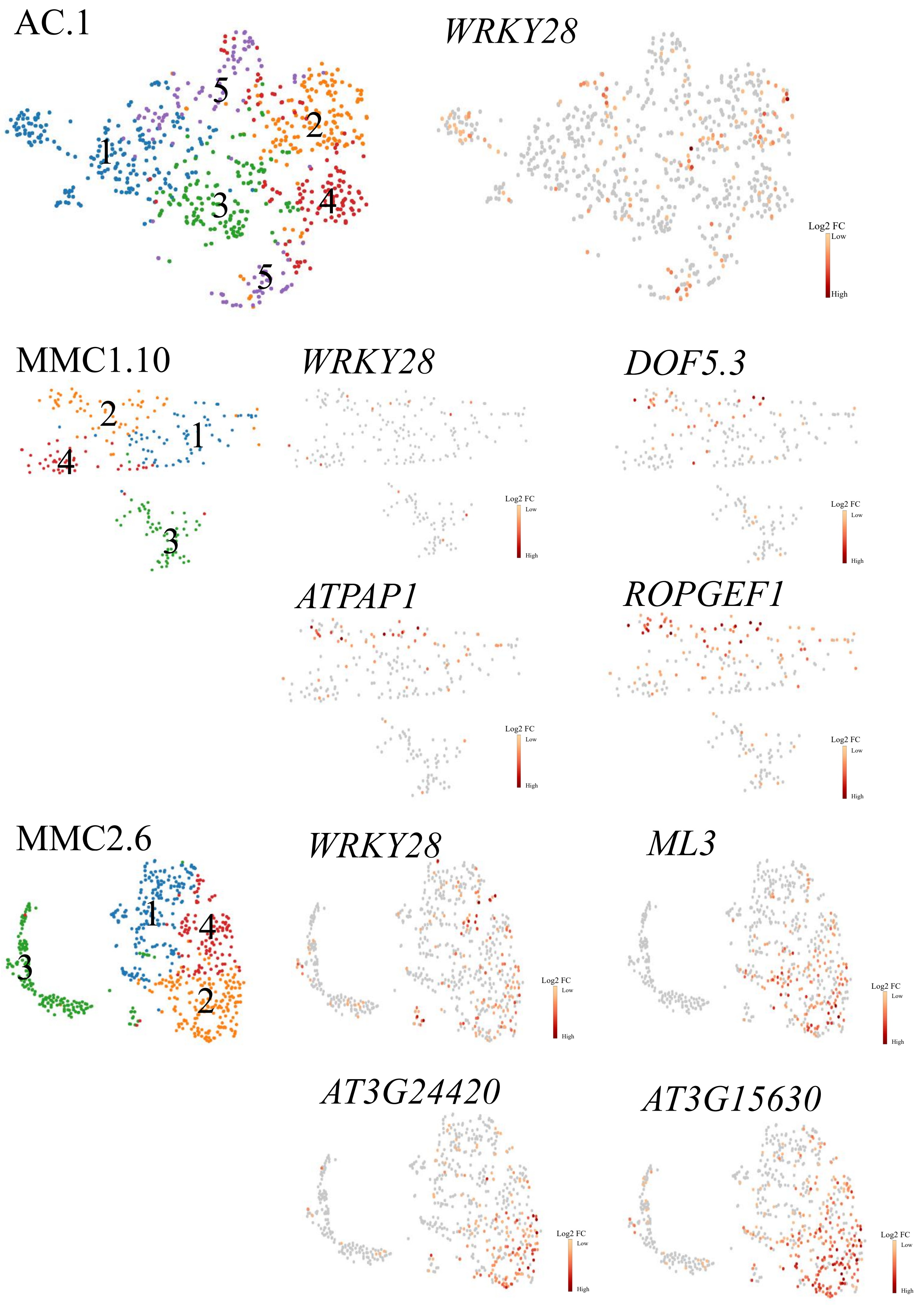

Supplementary Fig. 14. t-SNE visualization indicating the transcript accumulation for the marker gene *WRKY28* and the feature genes (*DOF5.3*, *ATPAP1*, *ROPGEF1*, *ML3*, *AT3G24420* and *AT3G15630*) of the sub-epidermis nucellar cell sub-clusters in individual cells of the AC.1, MMC1.10 and MMC2.6 cell clusters. Color intensity indicates the relative transcript level (Log2 FC) for the indicated gene in each cell.

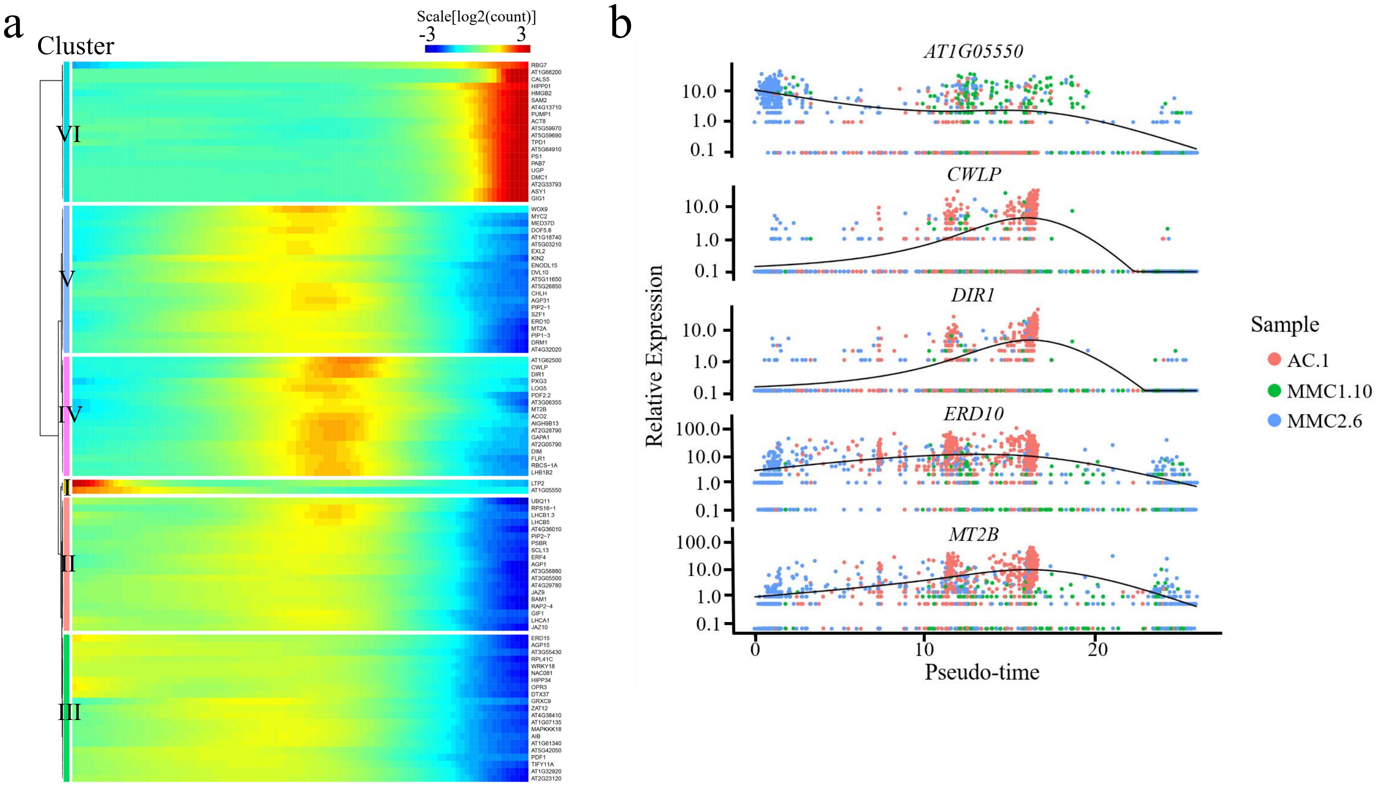

Supplementary Fig. 15. Gene expression waves of the top 100 DEGs across pseudotime.

a, Expression heatmap of the top 100 DEGs across pseudotime. The horizontal coordinate represents quasi-chronological order, and the vertical coordinate represents one gene per row. Each column represents the average expression value under the current cell state. Rows were grouped based on similarity of gene expression, resulting in the six clusters indicated at the left. Color bar indicates the relative expression level.

b, Expression values of the top five DEGs along the pseudotime axis. The abscissa represents the quasi-chronological order, and the ordinate represents the relative expression value of genes. The black line denotes the smoothed average expression. Cells are colored by sample identity.

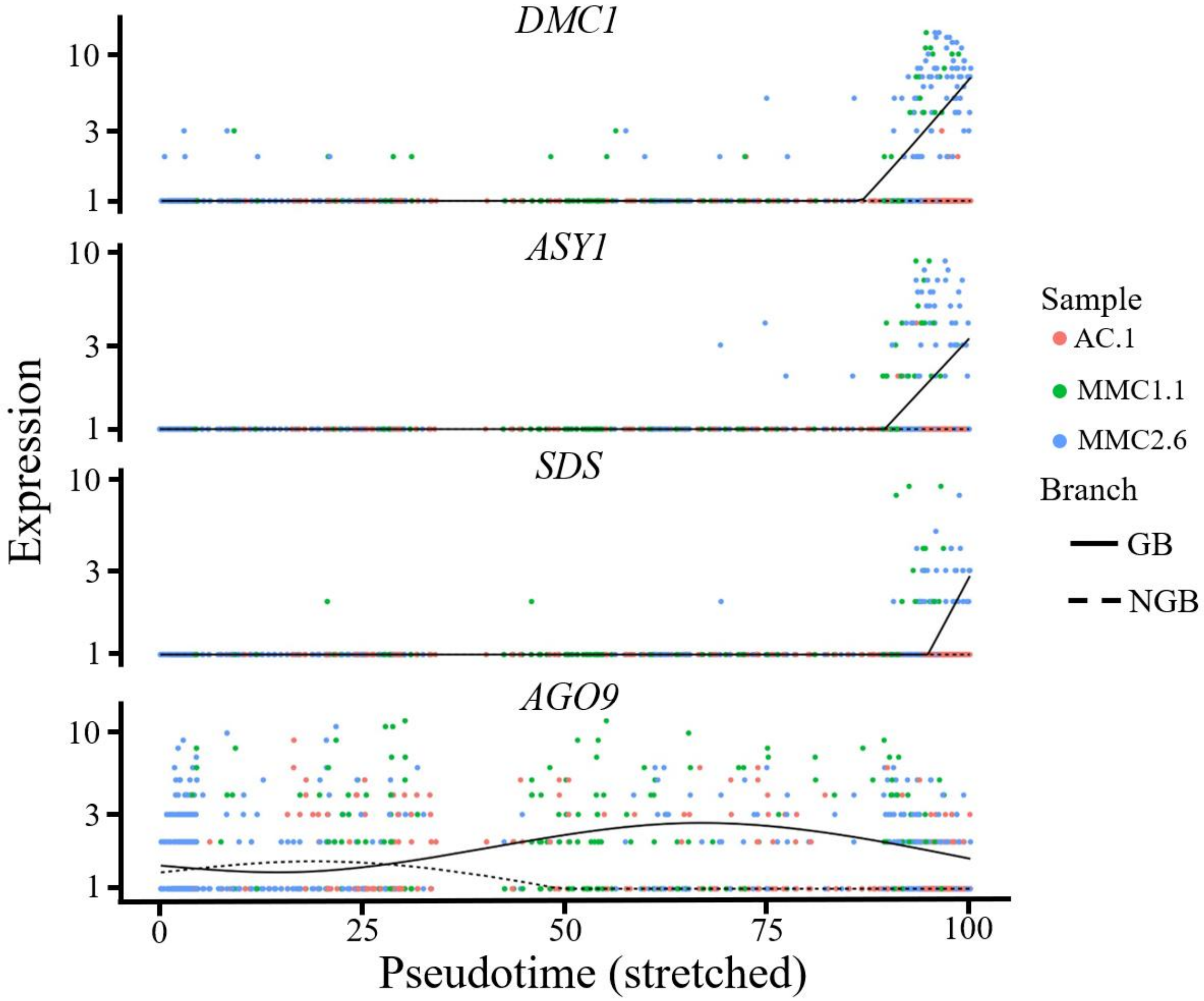

Supplementary Fig. 16. Expression of the known female germline marker genes *DMC1*, *ASY1*, *SDS* and *AGO9* along the two primary branches ordered in pseudotime. The abscissa represents the quasi-chronological order, and the ordinate represents the relative expression value of genes. Solid lines represent expression in cells in the germline branch (GB); dashed lines represent expression in cells in the non-germline branch (NGB). Cells are colored by sample identity.

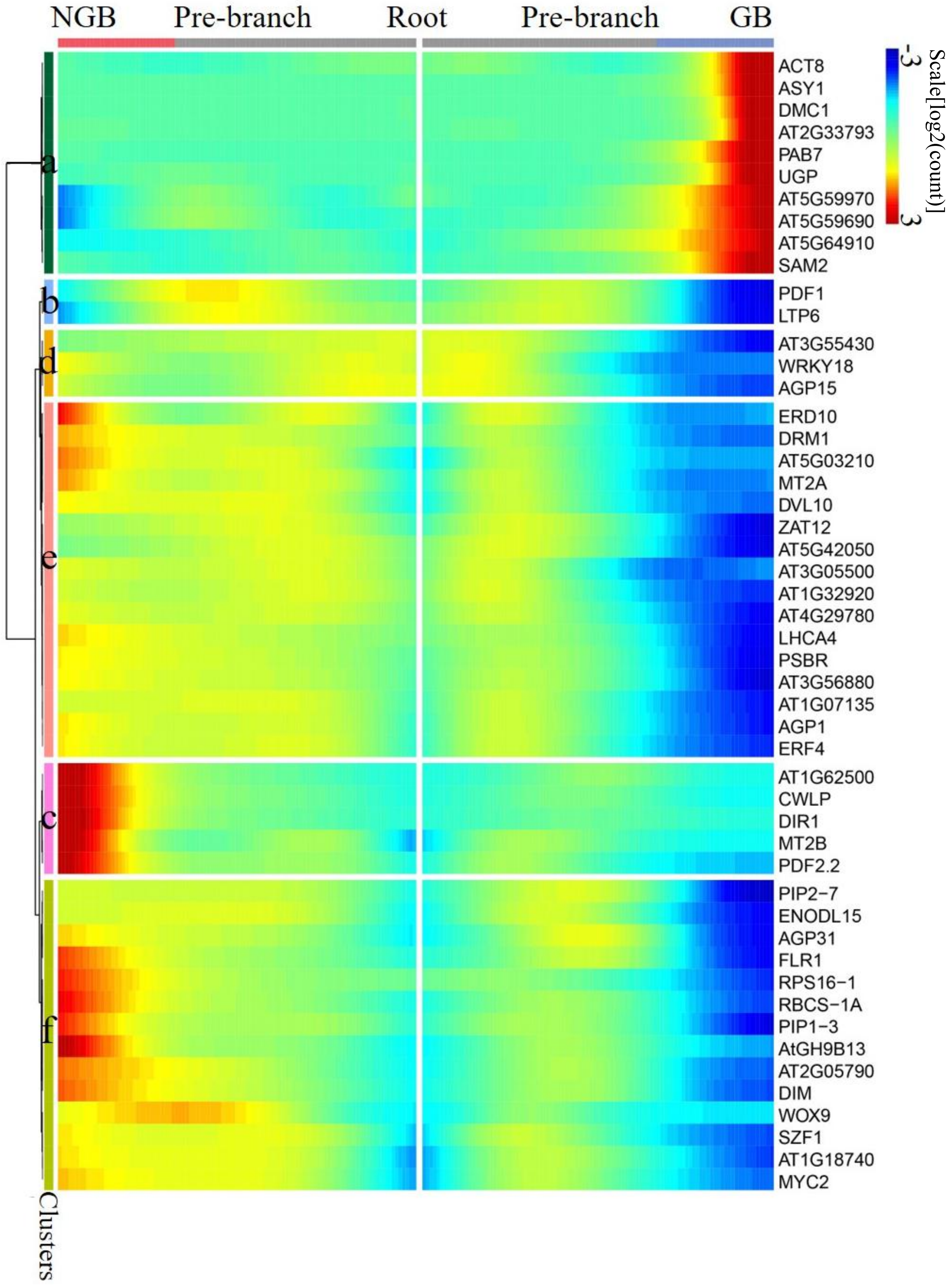

Supplementary Fig. 17. Branched heatmap showing the top 50 DEGs with branch-specific expression patterns in pseudotime. The root of the tree is in the middle of the plot, and expression from the earliest cells to the non-germline cells of the NGB is shown progressing to the left, whereas the germline cell progression in the GB is shown progressing to the right of the root. Color bar indicates the relative expression level.

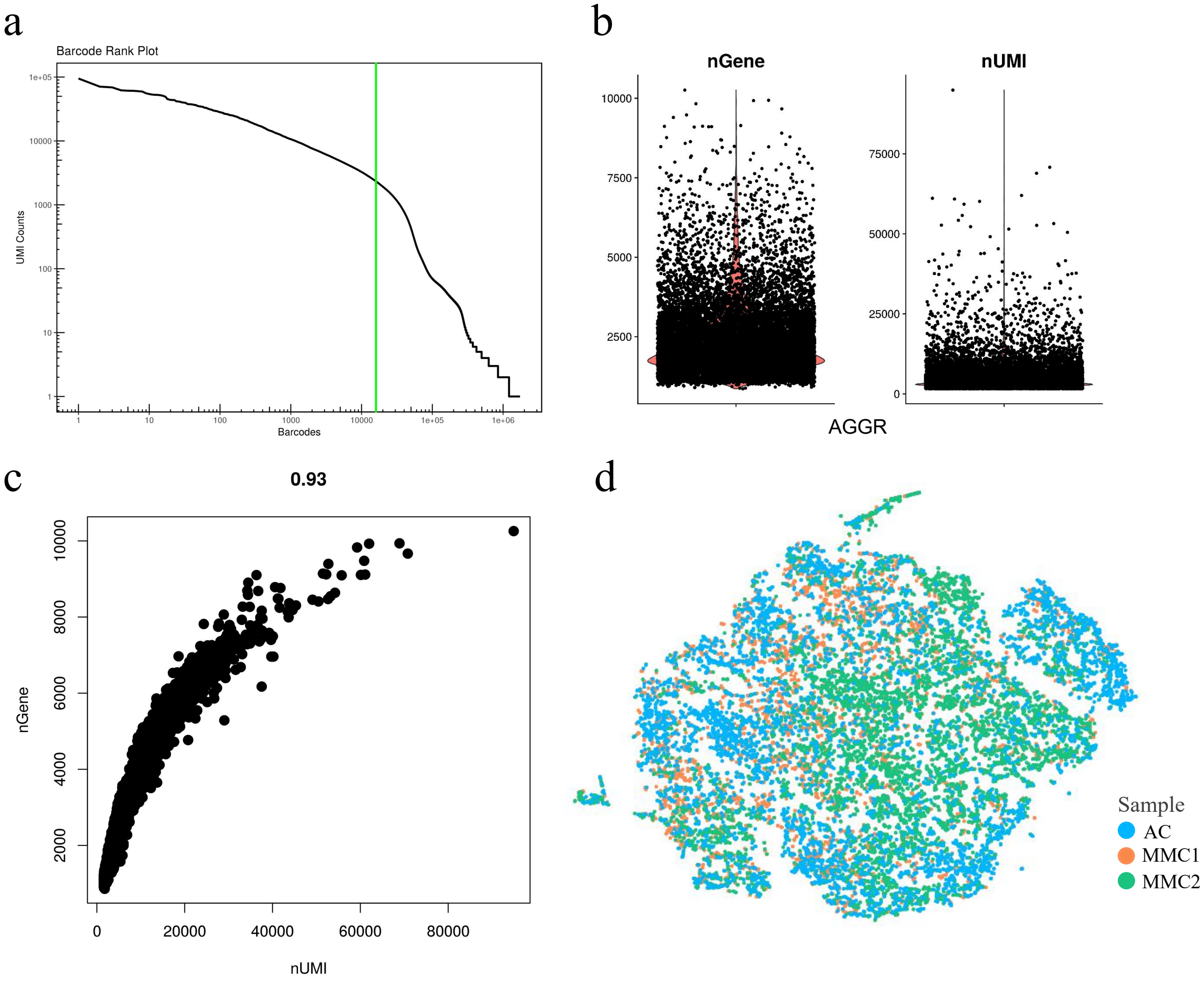

Supplementary Fig. 18. Evaluation of effective cell, distribution and correlation of genes and transcripts (UMIs) detected per cell integrated by AGGR pipeline.

a, Evaluation of effective cells integrated by the Aggregating Multiple GEM Groups (AGGR) pipeline. Horizontal axis: number of barcodes; vertical axis: number of UMIs. On the X-axis, barcode on the left side of the green marker line is used as the active cell, while barcode on the right side is used as the background.

b, Violin image of genes and transcripts (UMIs) in the integrated three samples by AGGR. The left panel shows the violin image of gene numbers detected in all the cells of the sample. The right panel shows the violin image of UMI numbers detected in all cells of the sample. The number of genes or UMIs contained in each cell is on the ordinate. Violin Plot is used to display the distribution status and probability density of multiple sets of data.

c, Correlation between gene number and transcript (UMI) number in the integrated three samples by AGGR. The UMI number of each cell is on the abscissa and the gene number is on the ordinate.

d, t-SNE projection of the three integrated samples by AGGR. Different colors represent different samples.

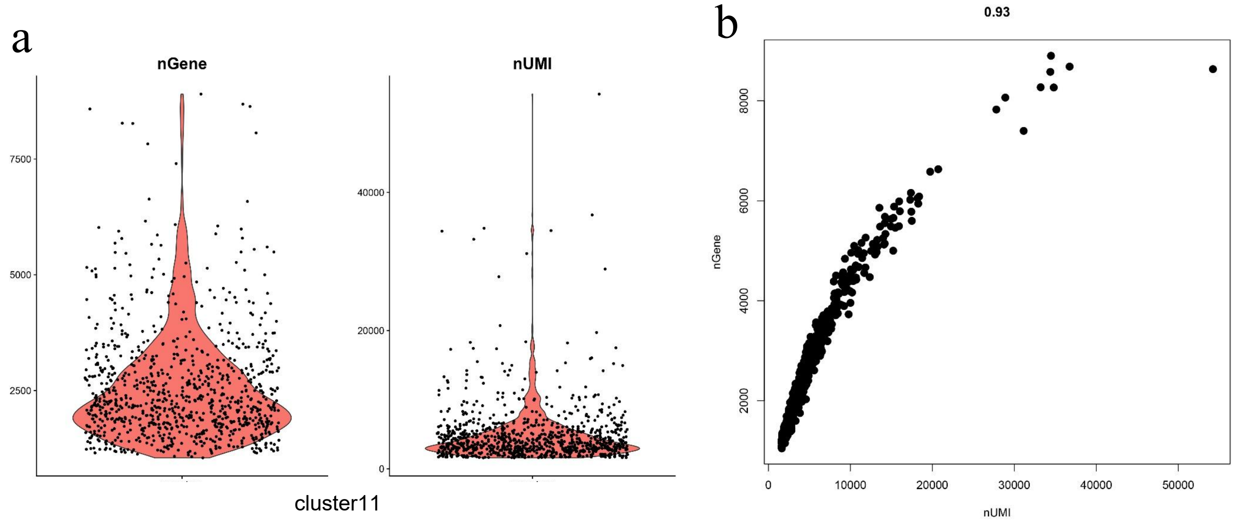

Supplementary Fig. 19. Genes and transcripts (UMIs) detected per cell of cluster 11.
a, Violin image of genes and transcripts (unique molecular identifiers, UMIs) in the cluster11. The left panel shows the violin image of gene numbers detected in all the cells of the sample. The right panel shows the violin image of UMI numbers detected in all cells of the sample. The x-coordinate is the sample name, and the y-coordinate is the number of genes or UMIs contained in each cell. Violin Plot is used to display the distribution status and probability density of multiple sets of data.
b, Correlation between gene number and transcript (UMI) number in the cluster11. The horizontal coordinate is the UMI number of a cell, and the vertical coordinate is the gene number of the corresponding cell.

AGGR.11

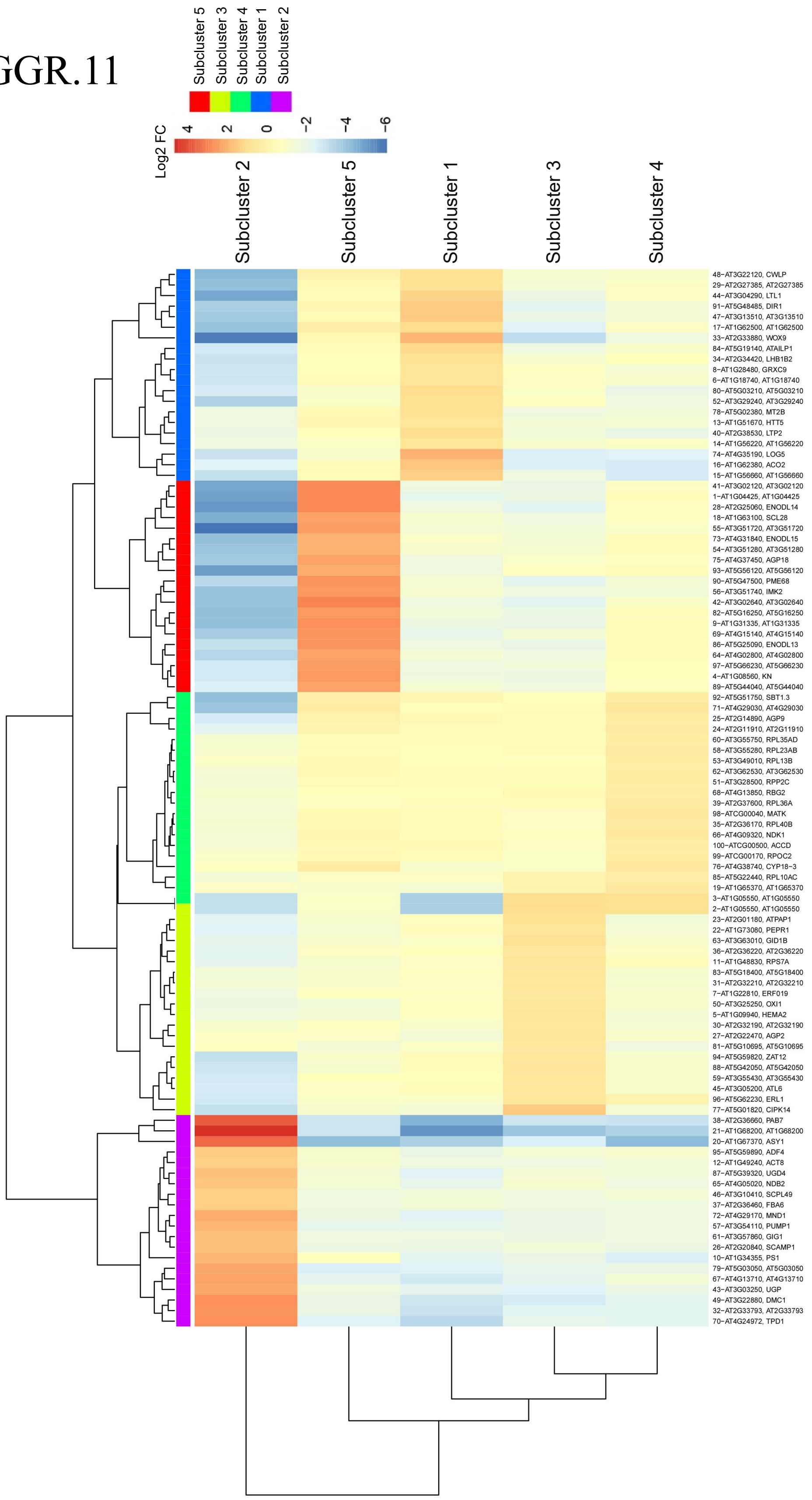

Supplementary Fig. 20. Heatmap displaying the expression of the top 20 feature genes of each cell cluster in the AGGR.11 cluster across the subclusters. Each column represents the cell cluster and each row represents one gene. The rainbow color bar indicates cell cluster identity. The red-to-blue color bar represents the average gene expression level (Log2 FC) from high to low.

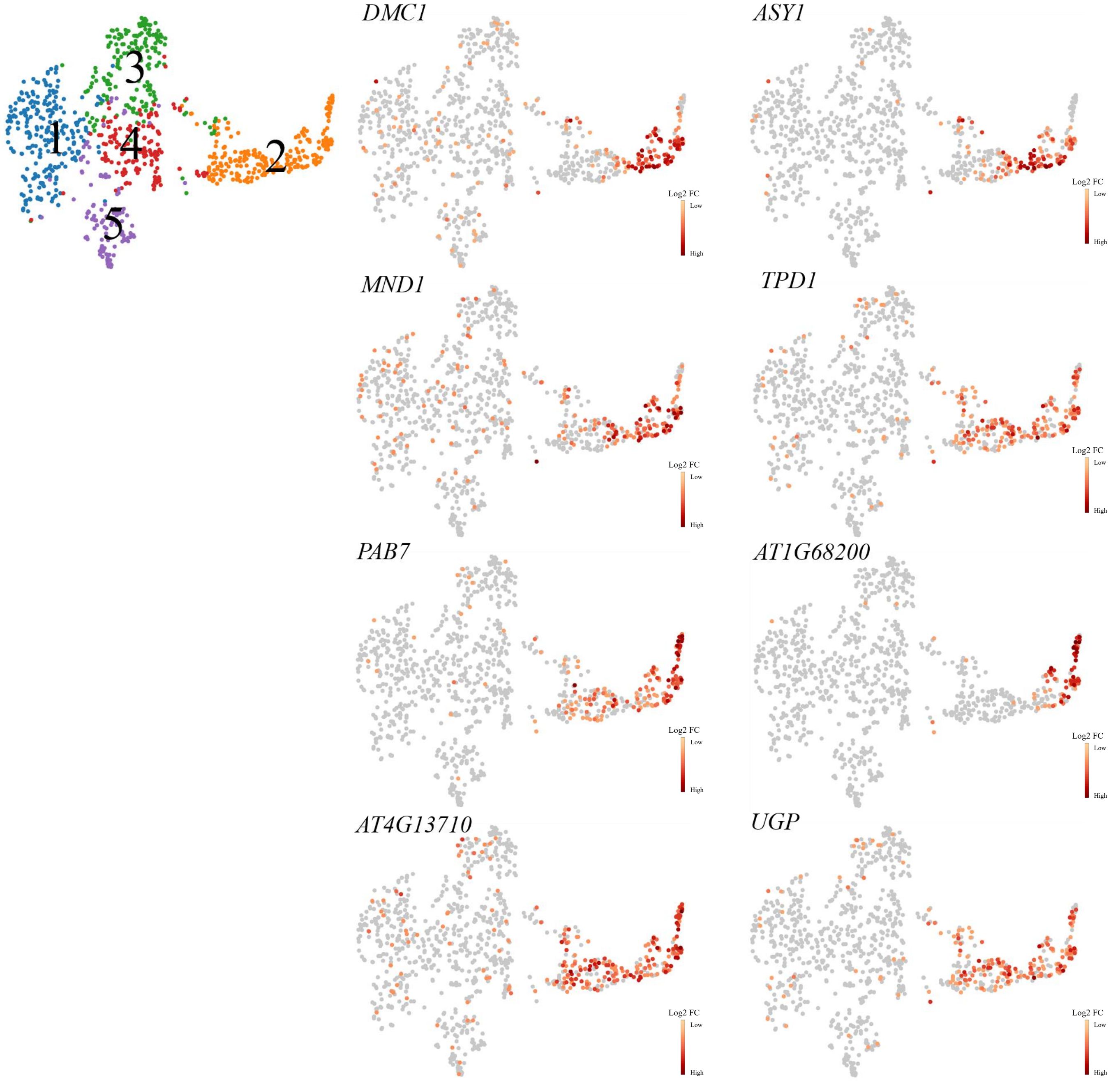

Supplementary Fig. 21. t-SNE visualization indicating the transcript accumulation for the feature genes (*DMC1*, *ASY1*, *MND1*, *TPD1*, *PAB7*, *AT1G68200*, *AT4G13710* and *UGP*) of the female germline sub-cluster in individual cells of the subcluster2 in AGGR.11 cell cluster. Color intensity indicates the relative transcript level (Log2 FC) for the indicated gene in each cell.
