# Supplementary material for: High-throughput single-cell transcriptomics reveals the female germline differentiation trajectory in Arabidopsis thaliana
Source: Commun Biol. 2021 Oct 1;4:1149. doi: 10.1038/s42003-021-02676-z (PMC8486858; doi:10.1038/s42003-021-02676-z)
Supplement: Supplementary file 2 — Description of Additional Supplementary Files [file 42003_2021_2676_MOESM2_ESM.pdf]

Description of Additional Supplementary Files

File name: Supplementary Data 1

Description: ScRNA-seq data metrics.

File name: Supplementary Data 2

Description: Number of cells in different cell clusters of AC, MMC1 and MMC2 stage samples and AGGR, number of different subclusters in AC.1, MMC1.10, MMC2.6 and AGGR.11 cell clusters.

File name: Supplementary Data 3

Description: Expression of known marker genes in the cell clusters in the AC, MMC1, MMC2 and AGGR samples and in the subclusters in the AC.1, MMC1.10, MMC2.6 and AGGR.11 cell clusters.

File name: Supplementary Data 4

Description: Expression of the top 20 feature genes in the cell clusters in the AC, MMC1 and MMC2 samples.

File name: Supplementary Data 5

Description: Expression and GO analysis of the feature genes of the subclusters in AC.1, MMC1.10 and MMC2.6 cell clusters.

File name: Supplementary Data 6

Description: Expression of the top20 feature genes in the subclusters in AC.1, MMC1.10, MMC2.6 and AGGR.11 cell clusters.

File name: Supplementary Data 7

Description: Expression of the feature genes in the AC.1, MMC1.10, MMC2.6 and AGGR.11 subclusters.

File name: Supplementary Data 8

Description: GO analysis of the feature genes in AC.1-subcluster1, MMC1.10-subcluster3, MMC2.6-subcluster3 and AGGR.11-subcluster2.

File name: Supplementary Data 9

Description: GO analysis of the feature genes in MMC1.10-subcluster 1, 2 and 4, MMC2.6-subcluster 1, 2 and 4, and AGGR.11-subcluster3.

File name: Supplementary Data 10

Description: Differentially expressed genes (DEGs) and GO analysis across pseudotime.

File name: Supplementary Data 11

Description: Differentially expressed genes (DEGs) with branch-specific expression and GO analysis across pseudotime.

File name: Supplementary Data 12

Description: Transcription factors (TFs) with branch-specific expression across pseudotime.

File name: Supplementary Data 13

Description: The Top100 Differentially expressed genes (DEGs) of AGGR.11 across pseudotime.

File name: Supplementary Data 14

Description: List of primers used in this study.
