# Supplementary material for: High-throughput single-cell transcriptomics reveals the female germline differentiation trajectory in Arabidopsis thaliana
Source: Commun Biol. 2021 Oct 1;4:1149. doi: 10.1038/s42003-021-02676-z (PMC8486858; doi:10.1038/s42003-021-02676-z)
Supplement: Supplementary file 17 — Reporting Summary [file 42003_2021_2676_MOESM17_ESM.pdf]

nature portfolio

Corresponding author(s): Yuan Qin

Last updated by author(s): Aug 30, 2021

Reporting Summary

Nature Portfolio wishes to improve the reproducibility of the work that we publish. This form provides structure for consistency and transparency in reporting. For further information on Nature Portfolio policies, see our Editorial Policies and the Editorial Policy Checklist.

Statistics

For all statistical analyses, confirm that the following items are present in the figure legend, table legend, main text, or Methods section.

n/a	Confirmed	
☐	☒	The exact sample size (*n*) for each experimental group/condition, given as a discrete number and unit of measurement
☐	☒	A statement on whether measurements were taken from distinct samples or whether the same sample was measured repeatedly
☐	☒	The statistical test(s) used AND whether they are one- or two-sided<br>*Only common tests should be described solely by name; describe more complex techniques in the Methods section.*
☐	☒	A description of all covariates tested
☐	☒	A description of any assumptions or corrections, such as tests of normality and adjustment for multiple comparisons
☐	☒	A full description of the statistical parameters including central tendency (e.g. means) or other basic estimates (e.g. regression coefficient) AND variation (e.g. standard deviation) or associated estimates of uncertainty (e.g. confidence intervals)
☐	☒	For null hypothesis testing, the test statistic (e.g. *F*, *t*, *r*) with confidence intervals, effect sizes, degrees of freedom and *P* value noted<br>*Give P values as exact values whenever suitable.*
☐	☒	For Bayesian analysis, information on the choice of priors and Markov chain Monte Carlo settings
☐	☒	For hierarchical and complex designs, identification of the appropriate level for tests and full reporting of outcomes
☐	☒	Estimates of effect sizes (e.g. Cohen's *d*, Pearson's *r*), indicating how they were calculated

Our web collection on statistics for biologists contains articles on many of the points above.

Software and code

Policy information about availability of computer code

Data collection

Protoplast digestion mixture was prepared fresh by adding 1.5% Cellulase R-10 and 0.2% Macerozyme R-10 (Yakult Company) to fresh protoplast buffer (1 M KCl, 1 M MgCl2, 1 M CaCl2, 0.1% BSA (Sigma Aldrich), 0.04% MES and 1% mannitol) and mixed thoroughly. Then, ovule primordia with placenta dissected from 60 pKNU::KNU-Venus floral buds at floral stages 9-10 were added to 2 ml of protoplast digestion mixture for digestion and incubated for 3 h with mild shaking at 100 rpm. The digestion mixture with protoplasts was filtered through a 40-µm-pore-size cell strainer, and the filtrate was centrifuged at 500 g for 5 min at room temperature. The supernatant was gently removed and resuspended with 1 ml fresh protoplast buffer. Protoplasts were validated under a Countstar instrument (Countstar Rigel S2). Single-cell RNA-seq libraries of fresh protoplasts were generated according to the protocol for the 10X Genomics Single Cell 3' Reagent Kit v2, and final library size and quality were assessed on an Agilent Bioanalyzer High Sensitivity chip. Then, libraries were quantified using the NEBNext Library Quantification Kit for Illumina. Finally, scRNA-seq library sequencing was performed on the NextSeq (Illumina) platform using the default parameters.

Data analysis

Firstly,The raw scRNA-seq data were analyzed and mapped to the TAIR10 Arabidopsis genome using the Cell Ranger pipeline 2.1.0 (10X Genomics). Secondly, PCA and t-distributed stochastic neighbor embedding (t-SNE) were used for dimensionality reduction and visualization analysis with the prcomp and Rtsne packages of the R software (Version 3.4.1). Thirdly, The Loupe Cell Browser (version 3.1.0) was performed graph-based clustering. Monocle finds DEGs (log2FC > 1, P < 0.05) with the differentialGeneTest function. GO biological process enrichment analysis was carried out using AgriGO (http://bioinfo.cau.edu.cn/agriGO/). Finally, Monocle 2 R package (version 2.8.0) was used to analyze the pseudotime trajectory of cell differentiation and the determination of cell fate. And the TFs differentially expressed were annotated in PlantTFDB (http://planttfdb.cbi.pku.edu.cn/). Gene regulatory network inference was calculated on the TFs using String (https://string-db.org/). Gene regulatory inference was filtered using different cutoffs on the parameter value. The gene correlation networks were visualized using Cytoscape, and the network topological parameters were obtained with NetworkAnalyzer

For manuscripts utilizing custom algorithms or software that are central to the research but not yet described in published literature, software must be made available to editors and reviewers. We strongly encourage code deposition in a community repository (e.g. GitHub). See the Nature Portfolio guidelines for submitting code & software for further information.

Data

Policy information about availability of data

All manuscripts must include a data availability statement. This statement should provide the following information, where applicable:

- Accession codes, unique identifiers, or web links for publicly available datasets
- A description of any restrictions on data availability
- For clinical datasets or third party data, please ensure that the statement adheres to our policy

ENA: PRJEB47244

Field-specific reporting

Please select the one below that is the best fit for your research. If you are not sure, read the appropriate sections before making your selection.

☒ Life sciences ☐ Behavioural & social sciences ☐ Ecological, evolutionary & environmental sciences

For a reference copy of the document with all sections, see nature.com/documents/nr-reporting-summary-flat.pdf

Life sciences study design

All studies must disclose on these points even when the disclosure is negative.

Sample size	Samples were generated from ovule primordia with placentas at developmental stages 2-I, 2-II and 2-III from stage 9-10 flower buds of the MMC marker line pKNU:KNU-Venus.The suspension volume of protoplasts met a density of 500-1,000 cells/μl.
Data exclusions	No data were excluded for the analysis.
Replication	For the analysis of the expression of pKNU:KNU-Venus in the ovule primordia and the germline specficiation phenotype of er-105 erl1-2 erl2-1 triple mutant, we performed three independent biological repetitions and each repetition included three floral buds.
Randomization	According to different morphological characteristics, 60 pKNU::KNU-Venus floral buds at floral stages 9-10 were collected, which is used to collect protoplasts of scRNA-seq. For the analysis of the expression of pKNU:KNU-Venus in the ovule primordia and the germline specficiation phenotype of er-105 erl1-2 erl2-1 triple mutant, we performed three independent biological repetitions and each repetition included three floral buds, so 9 floral buds of wild-type and er-105 erl1-2 erl2-1 triple mutant, respectively, for the germline specficiation phenotype.
Blinding	The investigators were blinded to group allocation during data collection and analysis.

Reporting for specific materials, systems and methods

We require information from authors about some types of materials, experimental systems and methods used in many studies. Here, indicate whether each material, system or method listed is relevant to your study. If you are not sure if a list item applies to your research, read the appropriate section before selecting a response.

Materials & experimental systems

n/a	Involved in the study	
☒	☐	Antibodies
☒	☐	Eukaryotic cell lines
☒	☐	Palaeontology and archaeology
☒	☐	Animals and other organisms
☒	☐	Human research participants
☒	☐	Clinical data
☒	☐	Dual use research of concern

Methods

n/a	Involved in the study	
☒	☐	ChIP-seq
☒	☐	Flow cytometry
☒	☐	MRI-based neuroimaging
